# Chemistry–mechanics–geometry coupling in positive electrode materials: a scale-bridging perspective for mitigating degradation in lithium-ion batteries through materials design

**DOI:** 10.1039/d2sc04157j

**Published:** 2022-12-08

**Authors:** David A. Santos, Shahed Rezaei, Delin Zhang, Yuting Luo, Binbin Lin, Ananya R. Balakrishna, Bai-Xiang Xu, Sarbajit Banerjee

**Affiliations:** a Department of Chemistry, Texas A&M University College Station TX 77843 USA https://twitter.com/sarbajitbanerj1; b Department of Materials Science and Engineering, Texas A&M University College Station TX 77843 USA; c Institute of Materials Science, Mechanics of Functional Materials, Technische Universität Darmstadt Otto-Berndt-Str. 3 Darmstadt 64287 Germany xu@mfm.tu-darmstadt.de banerjee@chem.tamu.edu; d Department of Aerospace and Mechanical Engineering, University of Southern California Los Angeles CA 90089 USA renukaba@usc.edu

## Abstract

Despite their rapid emergence as the dominant paradigm for electrochemical energy storage, the full promise of lithium-ion batteries is yet to be fully realized, partly because of challenges in adequately resolving common degradation mechanisms. Positive electrodes of Li-ion batteries store ions in interstitial sites based on redox reactions throughout their interior volume. However, variations in the local concentration of inserted Li-ions and inhomogeneous intercalation-induced structural transformations beget substantial stress. Such stress can accumulate and ultimately engender substantial delamination and transgranular/intergranular fracture in typically brittle oxide materials upon continuous electrochemical cycling. This perspective highlights the coupling between electrochemistry, mechanics, and geometry spanning key electrochemical processes: surface reaction, solid-state diffusion, and phase nucleation/transformation in intercalating positive electrodes. In particular, we highlight recent findings on tunable material design parameters that can be used to modulate the kinetics and thermodynamics of intercalation phenomena, spanning the range from atomistic and crystallographic materials design principles (based on alloying, polymorphism, and pre-intercalation) to emergent mesoscale structuring of electrode architectures (through control of crystallite dimensions and geometry, curvature, and external strain). This framework enables intercalation chemistry design principles to be mapped to degradation phenomena based on consideration of mechanics coupling across decades of length scales. Scale-bridging characterization and modeling, along with materials design, holds promise for deciphering mechanistic understanding, modulating multiphysics couplings, and devising actionable strategies to substantially modify intercalation phase diagrams in a manner that unlocks greater useable capacity and enables alleviation of chemo-mechanical degradation mechanisms.

## Abstract 2

The design of next-generation positive intercalation battery cathodes will leverage chemistry—mechanics—geometry coupling to mitigate stress, unlock more accessible storage capacity, and prolong cycle life.

## Graphical abstract

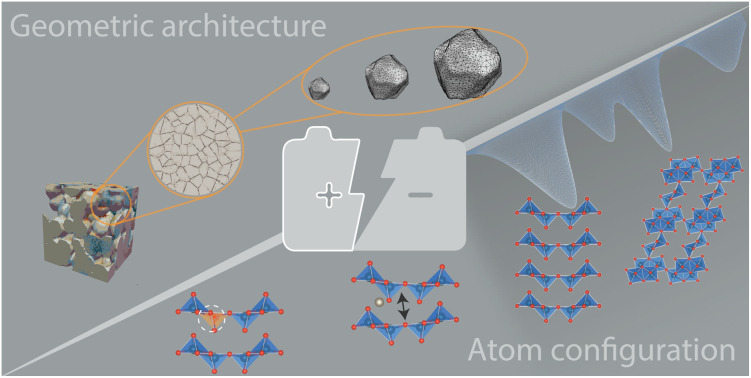

## 1. Introduction

Energy storage represents a critical bottleneck to the energy transition. Offsetting the intermittency of wind and solar sources necessitates access to high-capacity storage to reduce curtailment, provide load balancing, and enable energy arbitrage.^1^ Similarly, electromobility stands at a crossroads with vehicle electrification and has emerged as an increasingly urgent priority to mitigate the catastrophic environmental impact of fossil fuels.^2–4^ Lithium-ion batteries (LiBs) represent the dominant paradigm for electrochemical energy storage, which is largely a result of their high energy densities and the maturity of supply chains.^5^ However, emerging applications continue to demand performance beyond current metrics. The expansion of LiBs from consumer electronics to medium- and large-area formats underscores the need for high storage capacity and high-rate performance, as well as extended capacity retention.^6^ Furthermore, in the absence of robust recycling infrastructure and in light of the explosive growth of batteries with different form factors, ensuring long-term cyclability has assumed special importance from an environmental and resource scarcity viewpoint.^7^

The typical anatomy of a LiB comprises two current collectors interfaced with active electrode materials (positive and negative electrode materials), which facilitate charge/discharge functions *via* redox reactions, a liquid or solid lithium-ion electrolyte that enables ion transport between the electrode materials, and a porous separator. In its simplest form, the reversible operation of a LiB relies on the rocking-chair motion of Li^+^ between active electrodes, while the concomitant flow of electrons through an external circuit preserves electroneutrality. This construction gives rise to several interfaces, necessitates ion- and charge-carrier transport across scales, and requires orders of magnitude greater preference for insertion reactions over parasitic reactions such as oxygen evolution. Surface electrochemical reactions, bulk ion diffusion, redox reactions, and phase transformations can engender local gradients in chemical potential, which under various boundary conditions, beget and couple with structural distortions and stress gradients. Such multiphysics coupling can compromise the integrity of active materials, affecting accessible capacity as well as potentially triggering catastrophic failure.^6,8,9^

Understanding the fundamental scientific origins of battery degradation is of pivotal importance to extending the lifetime of commercial cells. Accounting for stress inhomogeneities represents a crucial step to the *a priori* design of high-performance electrochemical energy storage architectures. Principal degradation mechanisms of LiBs have been recently reviewed by Edge and colleagues.^9^ Capacity and power fade are traceable to mechanisms such as solid electrolyte interphase (SEI) layer formation, dendrite growth, structural disorder, interfacial (cohesive) delamination, and electrode fracture.^6,9,10^ However, the interplay and evolution of these mechanisms across length scales remain to be fully disentangled. A specific current gap we have sought to address in this article pertains to deciphering the atomistic origins of failure mechanisms and tracing their emergent complexity from the level of unit cells to single particles, aggregates, and electrode architectures across decades of length scales. We have further sought to go beyond the current focus on phenomenological descriptions to review emerging actionable strategies to mitigate degradation phenomena by leveraging chemistry–mechanics coupling.

Failure mechanisms have been characterized during and after battery, operation using a multitude of techniques that explore the interplay between lattice structure and anisotropic stresses within and beyond the limits of elastic deformation.^10,11^ Recent reviews have sought to develop empirical operating guidelines and practical strategies to limit degradation at the device and systems levels.^12,13^ In essence, the failure mechanisms of batteries are often traceable to local heterogeneities resulting from chemical potential gradients and structural imperfections that begin at the level of singular unit cells and compound across length scales.^14,15^ As such, it is imperative to develop an understanding of the coupling between compositional heterogeneities, atomistic structure, electronic structure, and the mesoscale architecture of individual battery components and their interfaces.

This work primarily focuses on high-capacity metal-oxide positive electrode materials with an emphasis on the composition V_2_O_5_ as a model system. This material affords a series of intercalation-induced phase transformations, multiple miscibility gaps between low- and high-lithiated phases and is characterized by a “rugged” energy landscape that allows for a variety of polymorphs to be stabilized with distinctly different diffusion pathways.^14^ Relative to state-of-art graphite anodes, which boast a reversible capacity of 372 mA h g^−1^, commercially used positive electrode materials have lesser accessible capacities (*ca.* 155 mA h g^−1^ for LiCoO_2_ and *ca.* 160–200 mA h g^−1^ for NMC), which limits the energy density of a cell.^6,16^ Particular emphasis is placed on degraded performance resulting from the components of porous electrode architectures, which are subject to intercalation-induced plastic deformation, fracture, and delamination.^6,9^ We seek to explore the thesis that harnessing multi-scale coupling between chemistry, mechanics, and geometry can address fundamental material limitations. While chemistry–mechanics coupling is often considered from the perspective of deleterious influences in battery materials, we aim to show that judicious materials design, leveraging this interplay, can be used to alter the kinetics and thermodynamics of diffusion phenomena to influence intercalation thermodynamics and kinetics and alleviate mechanisms of degradation.

This perspective comprises three primary sections. We begin with a conceptual discussion of chemistry–mechanics coupling in electrode materials. Bridging from crystallographic design principles to 3D porous electrode architectures, we connect solid-state chemistry design principles underpinning intercalation chemistry, specifically crystallographic design and site-selective modification approaches, to the emergent mesoscale structure of porous electrodes architectures by considering the coupling of solid mechanics with electrochemical processes across decades of length scales. Specifically, we have sought to link chemical design principles to degradation phenomena – mapping the role of materials design and processing to eventual functional performance. In doing so, we have delineated the role of operando spectromicroscopy probes of spatiotemporal compositional inhomogeneities, electronic structure theory, crystallographic theory, and phase-field modeling.

We next explore mechanics coupling across key electrochemical processes in positive electrodes. Specifically, we explore the interplay of mechanics with chemistry during electrochemical reactions at the electrode surface, bulk ion diffusion, and intercalation-induced phase transformations. In subsequent sections, we discuss recent findings on atomistic, crystallographic, and mesoscale structures as levers to modulate the interplay between electrochemistry and mechanics. This framework and linkages across these three key electrochemical processes represents a distinctive approach for considering the role of mechanics. Complementary to prior work that primarily focused on anodes, we highlight experimental work on cathode chemistries which enables the rich discussion of lattice strain, structural anisotropy, and the dynamics of phase transformations.

Finally, we emphasize actionable design strategies that leverage chemistry–mechanics coupling to mitigate failure mechanisms. This addresses a critical gap in the field that aims to bridge the gap from an observation of the problem to actionable design strategies.

## 2. A primer on electrochemistry–mechanics coupling in Li-ion batteries

Chemistry–mechanics coupling in battery materials considers the interplay between chemical, mechanical, and electric field driven forces during critical electrochemical processes.^6,17^ Given the topical nature of battery degradation, considerable attention has been paid to the deleterious consequences arising from the coupling of mechanics to electrochemical reactions.^18–21^ K. Zhao^22^ and co-workers have recently presented phenomenological descriptions of mechanics in LiBs; Y. Zhao *et al.*^23^ have reviewed the importance of studying electro-chemo-mechanics across length scales primarily through the lens of modeling. Given the efficient transfer of mechanical stresses at solid–solid interfaces, a growing emphasis on the intersection between chemistry and mechanics is evident in all-solid-state batteries (ASSBs).^17,24^ While the key developments showcased in this work are primarily focused on traditional LiB architectures comprising a liquid electrolyte, it is worth noting that the underlying principles of chemistry–mechanics coupling share several parallels with ASSBs.

### Origins of chemical heterogeneity and strain in electrode materials

The insertion and extraction of Li ions from an insertion host drive local lattice deformations and engender volumetric changes that induce stress when the expansion or contraction of the active material is constrained.^22^ At the atomistic level, local deformations with strong short range fluctuation often arise from ion insertion induced lattice size/structure change or from intralattice differences in lithium concentration which bring about anisotropic changes in lattice parameters^25^ and composition-dependent changes in the elastic and transport properties of the active material.^26^ Thus, to maintain a degree of registry across crystallographically misfit Li-rich and Li-poor regions, a strained interface is formed.^27^ Considering a two-dimensional model, the Li-rich region exerts a tensile in-plane force on the adjacent Li-poor domain, whereas an opposing compressive force is exerted on the Li-rich region. While the volumetric expansion of metal oxide cathodes (*ca.* 2–8%) is small in comparison to anodes (up to *ca.* 300%),^28^ even minor volumetric changes have been shown to yield plastic deformation in these typically brittle ceramic materials. For some cathode chemistries, dislocations are generated during repeated cycling, wherein a semi-coherent interface stabilizes the elastic misfit between phases.^29^ Stresses on the order of 300–500 MPa have been reported for V_2_O_5_ single particles^25^ and thin films.^28^ These intraparticle stresses are closely coupled to compositional heterogeneity which manifests in several ways. Arguably, the most recognizable origin of single-particle lithiation heterogeneity is the presence of phase separation stemming from intercalation-induced phase transformations.^27,30,31^ However, even when lithium insertion proceeds through solid solution formation, the presence of defects,^29^ particle geometry,^32^ surface facet termination,^33^ pre-existing strain,^25,34,35^ and crystalline anisotropy^36^ can drive a transient non-uniform distribution of Li-ions. These sources of single-particle chemical and strain heterogeneities are further amplified by the intrinsic directionality of electrochemical lithiation (initiated at the surface of the active material), manifested for instance as radial lithiation with a corresponding field of stress.

At the composite level, understanding stress and strain evolution first requires an appreciation of the diversity of constituents, their interfaces, and their long range spatial distribution within an electrode architecture comprising polydisperse redox-active particles embedded in a matrix of binder and conductive additives. In an ideal discharge/charge cycle, the entire volume of the electrode composite expands and contracts homogenously and cooperatively. In practice, the spatiotemporal dynamics of chemo-mechanical phenomena are governed by a host of factors that give rise to often discordant lithiation and mechanical dilation.^37,38^ For example, differences in thermally- or intercalation-induced expansion/contraction between the active material and the surrounding matrix have been shown to cause sufficiently high internal stresses to nucleate cracks at the interface between the particle and the matrix.^39^ Much like at the single-particle level, intercalation-induced stresses are exacerbated by heterogeneity. At the electrode level, particle size distribution,^40^ interparticle connectivity,^41^ electrode porosity,^38^ tortuosity,^42,43^ and differences in charge/discharge processes can all affect spatiotemporal variations in lithiation. Controlling these often-interdependent factors is imperative to balance cycling stability, energy density, and rate capability. The unifying feature that underlies the origins of stress/strain across length scales material is the coupling between chemical heterogeneity and mechanics, evidencing the importance of strategies that leverage this relationship to mitigate its unwanted implications to performance.

**Fig. 1:**
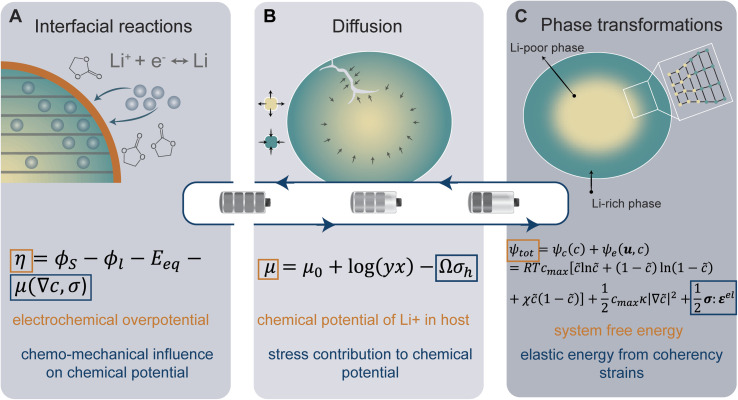
Schematic overview of chemistry–mechanics coupling in Li-ion batteries. The coupling between chemistry and mechanics is illustrated for, interfacial reactions, diffusion, and phase transformation processes in (A–C), respectively. A representative governing equation is provided below for each process and is accompanied by a simplified depiction of the terms linking mechanics (blue) with electrochemical phenomena (orange).

In the following sections, we highlight chemo-mechanical interactions in key mechanisms, including interfacial reactions, diffusion, and phase transformations, as illustrated in Fig. 1. We begin by discussing stress-regulated surface reactions. The cooperative interplay between stress and diffusion is introduced next. The final subsection highlights the rich chemo-mechanical considerations involved during intercalation-induced phase transformations, primarily associated with transition-metal-oxide positive electrodes. For each section, we describe the underlying mechanisms and provide a brief explanation of the governing equations at play (Fig. 1). Here, we aim to provide a holistic view of chemistry–mechanics coupling by bridging atomistic and electronic structure with microstructure and mechanics to give a concise guide to understanding this interplay in electrode materials.

### Chemo-mechanical coupling at the electrode–electrolyte interface

At the heart of a LiB are a series of multi-scale charge and mass transfer processes. The activation energy barrier associated with mass or charge transport is dynamically coupled to local stress states. At the interface between the electrolyte and the active material, a faradaic reaction occurs with charge transfer across the interface as depicted in Fig. 1A, generating a local current density which is represented by the well-known Butler–Volmer model of electrode kinetics.^44^ In concentrated solutions, the intrinsic rate of electron transfer at the electrode/electrolyte interface (*i.e.*, the exchange current density) is distinctly modified by stress. The modified Butler–Volmer equation shown in eqn (1) accounts for the combined electrical, chemical, and mechanical effect on the free energy and the activation energy for the surface charge transfer process.

1 *η* = *ϕ*_S_ − *ϕ*_l_ − *E*_eq_ − *μ*(∇*c*,*σ*)

Here, the overpotential, *η*, is a function of the electric potential in the electrode *ϕ*_S_, the electric potential of the electrolyte *ϕ*_l_, and the equilibrium potential *E*_eq_. *F* represents the Faraday constant. The chemical potential, *μ*, can be influenced by mechanical stress (*σ*), surface tension, and even the gradient of concentration (∇*c*) in the case of phase separation.^44,45^ In a simple isotropic case, the stress-driven change to the interfacial reaction can be accounted for by the product of the partial molar volume, *Ω*, and hydrostatic stress, *σ*_h_.^46,47^ Mechanical stresses affect the driving force for the surface charge transfer process by modifying the activation energy of redox reactions. This interplay can facilitate or impede charge transfer processes in a nontrivial manner. In the latter case, when the rate of ion insertion/extraction is much higher than bulk diffusion, saturation at the surface has been shown to shut down the intercalation reaction prematurely, limiting accessible capacity.^48^ Meanwhile, in a surface-nucleation-limited process, extrinsic factors such as particle size and geometry introduce asymmetric stress fields, which can promote spatial heterogeneity during surface reactions.^32^ This is especially prominent for nanometer-sized crystallites wherein surface stresses become more prominent, manifesting distinctive phase separation patterns rather than the conventional spherical shrinking-core phase morphology.^49^ In a later section elaborating on particle geometry effects, we show how understanding and controlling the interplay between sphericity, stress, and surface reactions can enable a modification of intercalation phase diagrams, spatiotemporal lithiation homogeneity, and accessible capacity.

### Cooperativity of stress and diffusion

The insertion of Li^+^ within a positive electrode material during (dis)charge drives local structural perturbations, volumetric expansion/contraction, and even phase transformations that deform the crystal lattice of active electrode materials. At increasing Li-ion concentrations, a reconfiguration of the atomic connectivity can be driven by both enthalpic and entropic factors. In this case, Li-ion intercalation drives softening/hardening of selective phonon modes, engendering a structural transformation.^50,51^ From the perspective of local atomistic structure, Li-ion intercalation creates anion–Li interactions and reduction of TM–O bonds, which thereby modify local coordination environments. In 2D materials, Li-ion insertion brings about a diminution of van der Waals' interactions, plane slippage, and altered layer stacking.^52^ Heterogeneous deformations due to internal lithiation gradients and structural incommensurability at solid–solid interfaces give rise to significant stresses that can contribute to degradation through the formation of defects such as dislocations,^29^ fracture, or delamination.^18^ Intercalation cathodes such as LiCoO_2_, Li_2_MnO_4_ (LMO), LiNi_*x*_Mn_*y*_Co_*z*_O_2_ (NMC), LiFePO_4_ (LFP), and Li_*x*_V_2_O_5_ may experience damage after only a few cycles, despite modest volume changes (*ca.* 2–8%), as a result of their characteristically brittle ceramic nature.^25,53–56^ Microcrack formation and growth is often a result of the initiation and accumulation of misfit dislocations.^57^ Such dislocations diminish interface mobility and can further contribute to fatigue crack initiation.^57,58^ The cracking-induced formation of new surfaces represents discontinuities in the displacement field, driving altered diffusion pathways and amplifying lithiation heterogeneities and their associated stresses.^59^ As such, the close coupling between mechanics and chemistry is clearly evident. Diffusion processes induce stress. In turn, stress contributes to the system's free energy through a modification of the chemical potential, whose gradient further drives diffusion.^22^Eqn (2) from the early work of Larche and Cahn^60^ demonstrates how the chemical potential of Li^+^ in an electrode is modified by volumetric strains induced during lithiation:

2 *μ* = *μ*_0_ + log(*yx*) − *Ωσ*_h_

Here, the *μ*_0_ + log(*yx*) term represents a reference chemical potential for Li^+^ in the host with concentration, *x*, and activity coefficient, *y*. The last term in eqn (2) captures the mechanical contribution in the form of a product between the partial molar volume *Ω* and the hydrostatic stress *σ*_h_ in the isotropic case, or in the general anisotropic case, expressed by the tensor product between the chemical distortion tensor and the stress tensor, *i.e.*, 
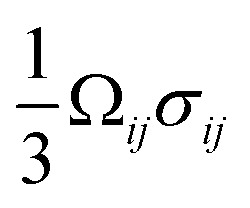
. In a simple isotropic case, assuming purely elastic behavior, stress gradients tend to homogenize Li-ion distribution, driving Li^+^ from Li-rich regions (under compression) to Li-poor domains (under tension).^22^

It is crucial to highlight that this principle of stress-driven diffusion is not limited to intercalation-induced stress fields. Stress gradients introduced during synthesis, as a result of epitaxial mismatch with a substrate, or through the application of external strain can be utilized as a tool to drive diffusion, even in the absence of electrochemical potential. This principle has been exemplified by the design of mechanical energy harvesters wherein bending-induced stress gradients drive a sufficiently large potential gradient to reversibly drive ion flux and generate electrical current.^35^ As a critical driving force for diffusion, engineering stress distributions to ensure that they remain within a crucial threshold for damage represents an opportunity to deterministically drive diffusion and access prolonged cycling.

### Coupling of chemistry and mechanics in phase-transforming electrodes

**Fig. 2:**
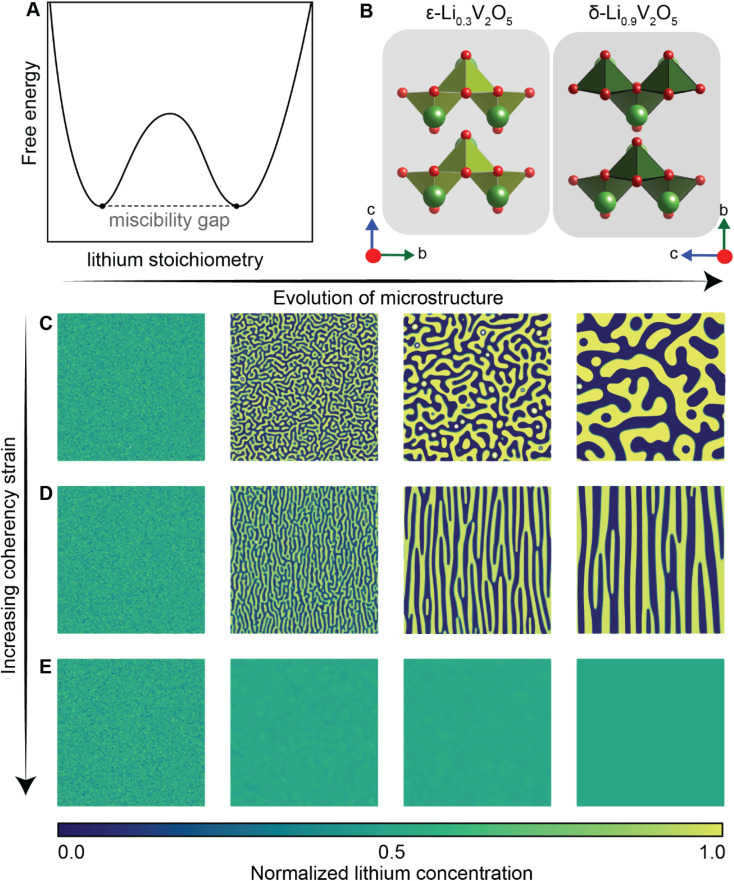
Chemistry–mechanics coupling in intercalation-induced phase transformations. (A) Simplified depiction of a free energy landscape with a double well potential that favors phase separation. The crystal structures of the ε-Li_0.3_V_2_O_5_ and δ-Li_0.9_V_2_O_5_ phase of Li_*x*_V_2_O_5_ phase diagram are depicted in (B) to illustrate the structural differences possible from lithiated phases separated by a miscibility gap. A microstructural evolution is modeled considering (C) 1% (D) 5% and (E) 20% volume changes between a Li-poor and Li-rich phase. At small volume changes (or lattice misfits) a two-phase separation motif is preferred. However, at larger volume changes (or lattice misfits) a solid-solution system is the energy minimizing microstructure. Panel (B) has been reproduced (adapted) with permission from Santos *et al.*,^25^ Copyright (2020) *Mater. Horiz.*

Of the fundamental mechanisms for accommodating inserted Li-ions, phase transformations represent a critical chemo-mechanical challenge for several high-capacity transition metal oxide positive electrode materials because of their implications on diffusivity, stress, and reversibility.^14,19,31^ In a simplified picture, the free energy function of such a system has a double-well feature (Fig. 2A) wherein phase separation is energetically preferred within the instability region.^51,61^ Within these regions, a system can minimize its total free energy, comprising both chemical and elastic terms as described by eqn (3), by phase separating into two structurally and compositionally distinct phases as exemplified in Fig. 2B using the ε- → δ-Li_*x*_V_2_O_5_ phase transition in the intercalation phase diagram of V_2_O_5_.3
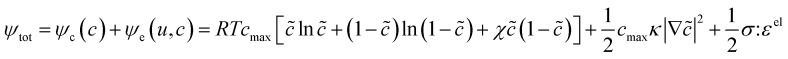
Here *c̃* = *c*/*c*_max_ is the normalized concentration; *R* and *T* denote the gas constant and temperature, respectively. Broadly, this free energy accounts for the gradient energy, 
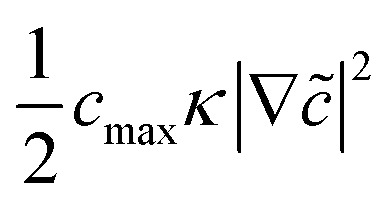
, which penalizes spatial changes in Li-ion composition, the homogenous bulk energy, *RT*c_max_[*c̃*ln *c̃* + (1 − *c̃*)ln(1 − *c̃*) + *χc̃*(1 − *c̃*)], which describes the free energy landscape of the material of interest, and the elastic energy, 
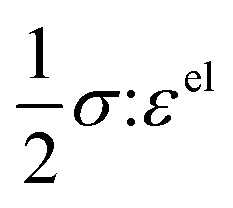
, which accounts for the coherency strain arising from the interface between the coexisting phases. Moreover, *χ* is a coefficient representing the interaction between two phases^25^ (beyond a diffusion-controlled phase transformation, the structural transformation and the ion transport should be regarded jointly in theoretical analysis). Indeed, at the interface between two incommensurate phases (schematically illustrated in Fig. 1C), coherency can be retained by distorting the equilibrium lattice parameters of both phases to maintain continuity (as opposed to an incoherent or semi-coherent interface where relaxation of this strain energy is accommodated by fracture or extended defects such as stacking faults or initiation of a misfit dislocation).^27,62^ Recent advances in phase-field methods capture the elastic energy effects arising from a polycrystalline electrode texture (by combining phase-field and phase-field crystal models),^63,64^ the presence of multiple crystal variants (by describing the free energy landscape using both composition and strain as order parameters),^65^ and model crack propagation (using a fracture phase-field framework).^66–68^

At equilibrium, in a chemo-mechanically coupled system, the energetic penalties associated with large concentration gradients and coherency strains arising from elastic misfits contribute to the resulting patterns of Li-rich and Li-poor domains. To conceptually illustrate this interplay, the microstructural evolution of a representative chemo-mechanical system is shown in Fig. 2C–E, for three simulations wherein lithiation induces 1%, 5%, and 20% volume changes, respectively. Here, the spinodal decomposition is solved using the standard Cahn–Hillard model to compute a time-dependent equilibrium microstructure. Fig. 2 shows that the thermodynamic driving forces for phase separation drive multiphase coexistence in the presence of relatively small coherency strains (Fig. 2C and D). At the same time, a solid solution is observed when the energetic penalty associated with the formation of an interface offsets any energetic gains from spinodal decomposition (Fig. 2E). In these simulations, the microstructure arises naturally from energy minimization, demonstrating that the interfaces between lithiated and unlithiated phases are dynamically oriented to reduce the total coherency strain.^25,27,69^ This interplay generates a rich variation of phase separation microstructures.^70,71^ Besides the continuum manifestation of phase-separation microstructures, the coupling between Li-diffusion and the underlying crystallographic texture of the host-electrode opens doors to designing intercalation electrodes.^63,64^

As briefly introduced above, not all interfaces retain their coherency in the presence of lattice mismatch.^62^ As is the case of semi-coherent interfaces, elastic misfit can be partially accommodated by defects that concentrate the lattice misfit into localized regions of incoherency.^72^ While relieving misfit strain in the presence of a phase boundary, defects often introduce local stress and inelastic strain fields, which can alter diffusion during subsequent cycling.^73^ For example, in Li_2_MnO_3_, dislocations originating at the interface between lithiated and delithiated phases have been shown to govern delithiation through a dislocation climb mechanism.^74^ Using a non-singular continuum dislocation approach, recently developed mechanically coupled diffusion models have represented the impact of dislocations as an eigenstrain that modifies the spatiotemporal concentration and diffusion of lithium ions.^75^

From a thermodynamics perspective, it is crucial to consider that point defects store energy in crystalline materials (proportional to the defect formation energy) and, thus, represent high-energy states in the lattice.^76^ In multiphasic intercalation systems, these high-energy states have been shown to act as nucleation points for phase transformations.^29^ By leveraging this relationship, one can envision an approach where defects are engineered before cycling to synergistically alter the nucleation and growth process to minimize stress and unwanted plasticity.^77^ Without adequate control, however, defects can be generated, dissipated, or even concentrated in such a way that a system's free energy and round-trip efficiency is detrimentally impacted. This has been observed in lithium-rich layered oxides where the origin of voltage fade has been ascribed to the accumulation of a dislocation network, which raises the Gibbs free energy of the system.^78^ Whether intrinsic, intercalation-induced, or synthetically tailored, engineering resilient and efficient energy storage devices in the presence of defects remains both a challenge and an opportunity in the design of battery electrodes.

The unifying feature that underlies the different chemo-mechanical phenomena observed at coherent and semi-coherent interfaces is the coupling between lattice misfit and intercalation. In a later section, we discuss materials design strategies that directly target lattice misfits to design stress-minimizing microstructural patterns.^71,79^ In addition to the interfacial penalty that offsets the thermodynamic driving force for phase separation, other operating conditions such as electrochemical kinetics, mechanical constraints, electrode particle shape, and geometry are shown to affect multiphasic lithiation patterns.^80–82^ The interplay between strain and phase-separation phenomena is a clear opportunity to fundamentally alter intercalation phase diagrams and to engineer low-stress phase separation patterns in a manner that allows for complete reversibility and minimal voltage hysteresis.^71^ As shown in the subsequent sections, strain engineering is being applied across multiple length scales to engineering bulk properties as well as individual lattice transformation pathways.

### Deciphering mechanistic understanding to develop design strategies for mitigating performance degradation

**Fig. 3:**
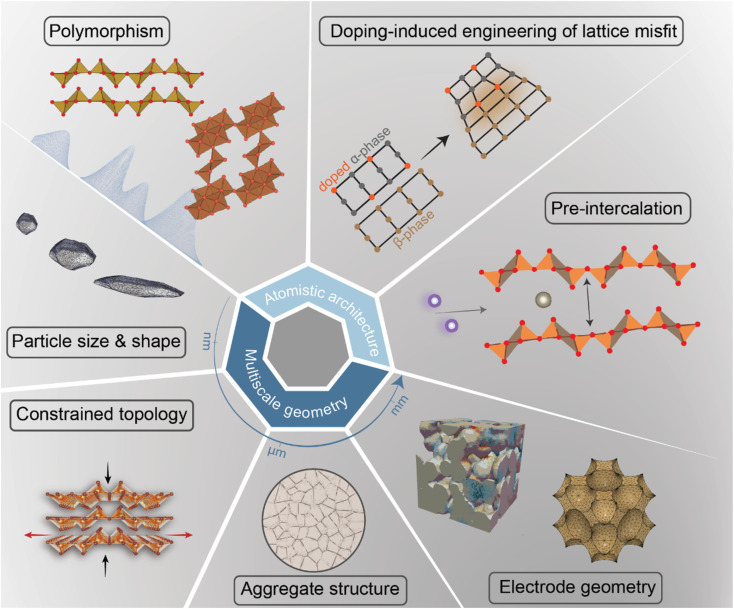
A diverse palette of levers by which fundamental chemistry–mechanics coupling mechanisms in cathode materials can be altered to control intercalation-phase transformations, mitigate stress accumulation,^25^ and prolong cycle life.

In intercalation positive electrodes, cation (de)insertion and diffusion in the host structure induces a wealth of chemo-mechanical phenomena, including constrained deformations and distortive phase transformations with substantial implications for stress inhomogeneities. Meanwhile, the resulting mechanical stresses make a nontrivial contribution to a system's free energy, thereby regulating surface reactions, bulk diffusion, and phase-transformation processes in positive electrode materials (Fig. 1). Without intervention, this feedback loop often manifests in growing stochasticity,^83^ increasing spatiotemporal compositional inhomogeneities, stress accumulation, and a resulting loss of electrochemical and mechanical stability of the active material. In a chemistry–mechanics-informed approach to materials design, the same principles linking electrochemistry to mechanics can be leveraged to design electrodes with pre-determined intercalation characteristics spanning across multiple length scales that minimize the accumulation of stress. Having begun with an emphasis on the underlying mechanisms linking chemistry and mechanics, we now shift our focus to tangible strategies that make use of pre-determined geometric and atomistic structure perturbations to facilitate diffusion, enhance charge and mass transport, and fundamentally modulate the phase transformation characteristics of insertion electrode materials as summarized in Fig. 3.

## 3. Shaping-up active electrodes: unlocking performance through geometric design

A typical Li battery electrode is characterized by a hierarchical structure wherein agglomerations of single crystallites of the active material are combined with polymeric binders and conductive additives to form a porous electrode architecture.^84^ While the performance characteristics of an intercalation electrode are undeniably linked to its chemistry, geometric differences across electrodes sharing an identical composition can lead to striking differences under cycling conditions. In this perspective, the discussion of geometry encompasses three elements: a geometric origin, an observable effect, and a mechanism. At the single-particle level, for example, particle dimensions (origin) are linked to ionic transport (effect) through a change in diffusion path length and surface stresses (mechanism).^40^ At the level of aggregates, the spatial orientation and shape of primary particles is connected to microstructural heterogeneity through distinct mechanisms of particle ensemble lithiation.^41^ In thick porous electrode structures, areal capacity can be improved significantly by modulating ion transport pathways through control of tortuosity and porosity.^42,43^ As such, the definition of geometric design here describes an approach wherein the physical attributes of the active material are tuned, across one or more length scales, without altering composition or surface chemistry to achieve a desired electrochemical improvement. This section is broadly organized into individual-particle-level and electrode-level geometry ideas, with a discussion of bridging scales throughout to not lose sight that the two are ultimately interconnected.

### Particle geometry: mitigating transport limitations through nanometer-sized crystallite geometries

Previous studies have shown that reducing the dimensions of primary electrode particles shortens diffusion pathways and increases the surface area-to-volume ratio to maximize the electrode–electrolyte interface and improve ionic transport.^40,81^ As a result, attempts to offset the transport limitations in several commercialized positive electrode materials, such as LiFePO_4_ (ref. 85) and LiCoO_2_ (ref. 86) include a reduction in particle size. While the link between particle size and transport kinetics is now well appreciated, the influence of primary particle size and shape on intercalation phase diagrams has only recently been leveraged to circumvent coherency strains and their associated implications for degradation.^81^

**Fig. 4:**
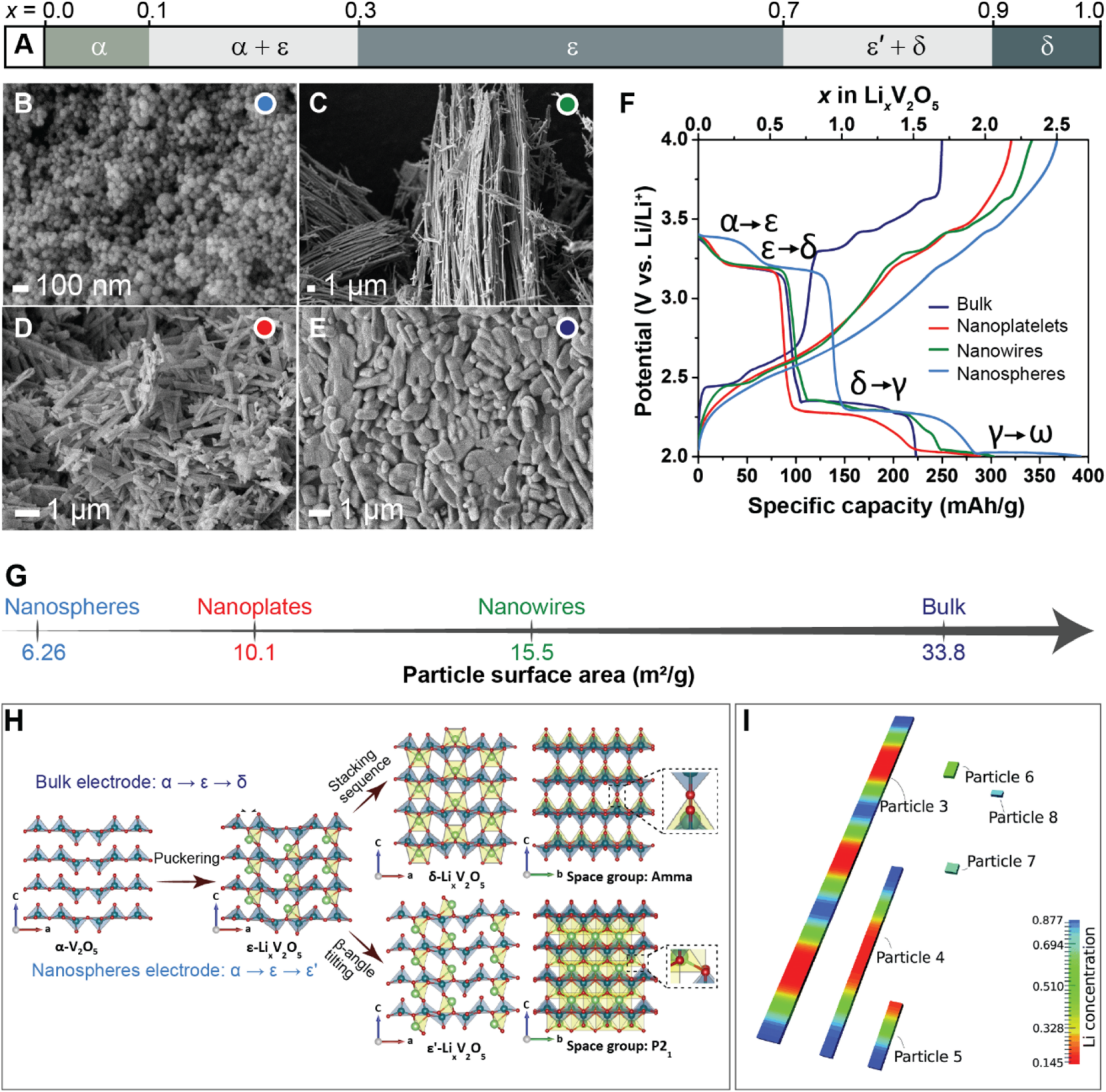
The interplay between primary particle geometries and phase transformations. (A) Simplified phase diagram showing evolution of lithiation-induced Li_*x*_V_2_O_5_ phases. Scanning electron microscope (SEM) images of (B) nanospheres, (C) nanowires, (D) nanoplates, and (E) micron-sized “bulk” platelets of orthorhombic α-V_2_O_5_; (F) discharge/charge profiles of α-V_2_O_5_ particles at C-rate of C/5 for the first cycle between 2.0–4.0 V; (G) calculated surface area of α-V_2_O_5_ powders by Brunauer–Emmett–Teller (BET) analyses. (H) Shows the crystal structures of α-V_2_O_5_, ε-Li_*x*_V_2_O_5_, ε′-Li_*x*_V_2_O_5_ and δ-Li_*x*_V_2_O_5_ while contrasting the profoundly different phase evolution observed from bulk and nanosphere electrodes. (I) Modeling of phase separation across particles with different size. The smaller particles are lithiated at higher rates due to larger surface-area-to-volume ratios, (H) has been reproduced (adapted) with permission from Luo *et al.*,^81^ Copyright (2022) *Nat. Mater.* Panel (I) has been reproduced (adapted) with permission from Zhao *et al.*,^180^ Copyright (2017) *RSC Adv.*

To illustrate the multi-faceted role of particle geometry on electrochemical performance, the shapes of several α-V_2_O_5_ samples have been synthetically tailored to access nanosphere (0D), nanowire (1D), nanoplatelet (2D), or micron-sized platelet “bulk” (3D) geometries, which are further distinguished based on their lateral dimensions and surface area, as shown in Fig. 4B–E and G, respectively. In the α-V_2_O_5_ system, pronounced interparticle and intraparticle lithiation gradients have been observed on account of several miscibility gaps in its intercalation-induced phase diagram plotted in Fig. 4A. This cathode system embodies the quintessential electrode dilemma wherein a host of desirable characteristics are undermined by a few intrinsic limitations – in this case, poor electrical conductivity and a series of intercalation-induced phase transformations.^14^ To delineate the effect of particle geometry in this intercalation electrode, galvanostatic discharge/charge profiles are plotted within the same working potential window in Fig. 4F. During discharging to 2 V at a C-rate of C/5, a size dependence on specific discharge capacity is observed. The nanosphere morphology shows the highest specific capacity at 392 mA h g^−1^, followed by the nanowires and nanoplatelets at 303 and 289 mA h g^−1^, respectively. In this series, the bulk material shows the lowest discharge capacity, which is consistent with the presence of higher internal resistance in the bulk particles through longer diffusion pathways and a smaller specific surface area.^40^ The absence of a voltage plateau in bulk particles at 2.03 V, corresponding to the γ-Li_*x*_V_2_O_5_ to ω-Li_*x*_V_2_O_5_ phase transformation, evidences an incomplete transformation to the highly lithiated ω-Li_*x*_V_2_O_5_ and points toward a size effect on overpotential. By scaling down particle size, diffusion pathlengths are truncated, which further translates to reduced electrode-level phase inhomogeneities.

In recent work, a comparison between two α-V_2_O_5_ electrodes contrasting bulk particles and nanospheres (NS) shown in Fig. 4 reveals a clear dependence of crystallite geometry on the spatiotemporal evolution of lithiated phases in this system.^81^*Operando* synchrotron X-ray diffraction (XRD) tracking phase evolution and energy dispersive X-ray diffraction (EDXRD) examining slices of the positive electrode at different distances from the separator demonstrate that phase transformations for the bulk material follow the well-documented α- ↔ ε- ↔ δ-phase sequence.^87,88^ In contrast for NS-structured V_2_O_5_, the transformation between ε/δ is supplanted by solid-solution formation with preferential stabilization of an ε′-Li_*x*_V_2_O_5_ phase appearing at *x* = 0.75 as depicted in Fig. 4H. For the spherical nanoparticles, a more uniform current density, deriving from particle shape, combined with a reduction of bulk transport limitations resulting from particle size, appears to engender homogenous lithiation.^41^ In V_2_O_5_, the interfacial mismatch at ε/δ interface has been shown to be relatively large, which incurs a significant elastic penalty.^25^ These elastically stressed interfaces are energetically unfavorable in nanometer-sized electrode particles (because of competing surface and bulk energy terms), and thus the lattice transformation pathway is modified.^71^ Indeed, these results are consistent with size-dependent heterogeneity and elimination of phase boundaries in particles <*ca.* 40 nm predicted by phase-field modeling (Fig. 4I).^41^ These results show that a reduction in particle size and increase in sphericity can fundamentally change intercalation-induced phase diagrams. The potentially beneficial aspects of this geometry-induced change have been clearly illustrated by multibeam optical stress sensor measurements (MOSS) and phase-field modeling, which together demonstrate that a reduction in phase heterogeneities for smaller and more uniform particles directly translates to a decrease in stress accumulation during cycling.^81^ Even when biphasic lithiation regimes are retained upon a change in geometry, a resulting change in the balance between reactive surface area, bulk volume, and dimensionality of transport drives profoundly different lithiation patterns with distinct stress gradients, demonstrating the importance of tuning geometry at the single-particle level.

However, it is worth noting that a reduction in particle size is not a panacea; balancing particle dimensions to enable improved electrochemical performance requires an understanding of the associated trade-offs since an increased surface area results in a greater proclivity for side reactions, such as oxygen evolution and the formation of a thicker SEI.^40,89^ To this effect, an increasing body of work has focused on deciphering the particle-dependent evolution of lithiation heterogeneities and the rational geometric design of individual particles and resulting porous architecture assembled from such building blocks.^40,90–92^

**Fig. 5:**
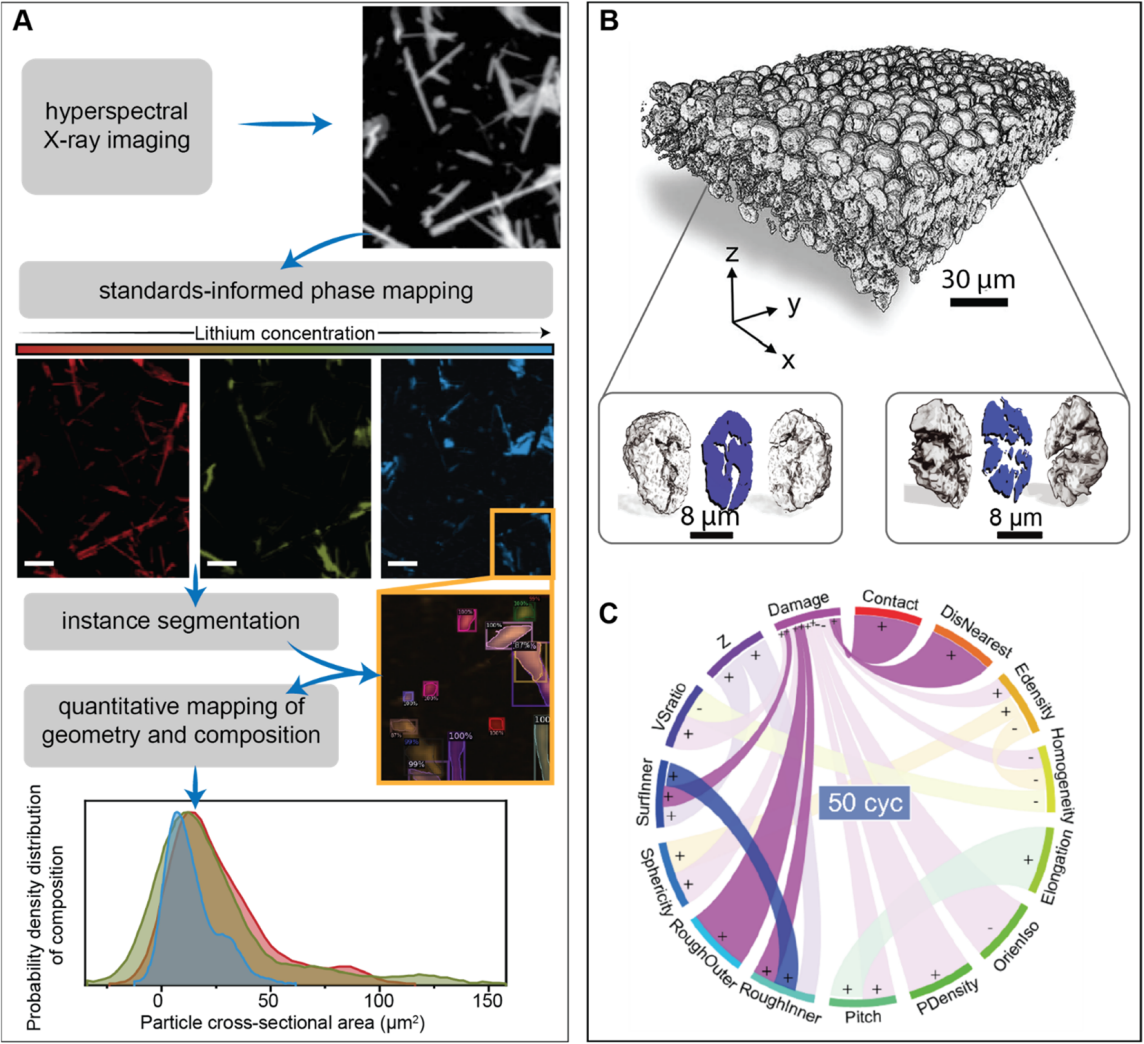
Applications of high-dimensional imaging techniques to study the role of particle geometries in battery performance. (A) Schematic representation of a deep-learned algorithm developed to perform instance segmentation of a polydispersion of nanoparticles whose geometry and composition have been mapped by X-ray hyperspectral imaging. Reprinted (adapted) with permission from Santos *et al.*,^96^ Copyright (2022) *Patterns*. (B) Visualization of a composite NMC electrode and its primary particles, resulting from X-ray holotomography measurements. The correlation of 15 attributes in illustrated by the circular plot in (C) The “+” and “−” symbols describe a positive or negative correlation between attributes, respectively. Panels (B) and (C) were reprinted (adapted) with permission from Li *et al.*^95^ Copyright (2022) *Science*.

Unravelling the interplay between particle geometry and cell performance is a multifaceted problem spanning multiple length and time scales. Mechanistic understanding of this intricate correlation has been significantly aided by scale-bridging characterization efforts that often involve the confluence of high-dimensional datasets and data science techniques.^93–95^Fig. 5A showcases a deep learning model based on the Mask-RCNN algorithm, which performs the image segmentation task on a polydisperse ensemble of positive electrode particles imaged by X-ray spectromicroscopy.^93,96^ The combination of this segmentation algorithm with chemical imaging enables the colocalization of morphology with state-of-charge and phase coexistence across several length-scales.^96^ The intention of this type of workflow is to pinpoint how the chemo-mechanical behavior of single-particles relates to their geometry and influences ensemble reaction heterogeneities.

One exemplary demonstration, from Li *et al.*, imaged thousands of LiNi_0.8_Mn_0.1_Co_0.1_O_2_ particles, upon repeated cycling, using phase contrast from X-ray nano-holotomography.^95^ In line with the approach of scale-bridging characterization, the spatial resolution of this technique not only enables a detailed visualization of the composite cathode but also damage within primary NMC particles, as shown in Fig. 5B. Here, a detailed analysis of various physical descriptors is enabled by a neural network – based particle identification method. As illustrated in Fig. 5C, the pairing of high-dimensional imaging with machine learning facilities an interpretation of how particle attributes are either positively or negatively correlated. Interestingly, their findings suggest that certain primary particle attributes, such as sphericity, have a stronger influence on damage at the early stages of cycling, whereas the interplay between particles (detailed in a subsequent section) becomes more important during later cycles.

In summary, shortened diffusion pathways facilitate the homogenous distribution of an intercalated species, driven by either stress or chemical potential, which alleviates the adverse effects of heterogenous deformation. While an increase in the surface-area-to-volume ratio of spherical nanometer-sized electrode particles lowers the energetic barriers for nucleation, the increased reactivity of side reactions should be balanced against the benefits of improved interfacial reaction kinetics and more favorable tortuosity. The confluence of phase-field modeling, artificial intelligence (AI)/machine learning (ML)-aided scale-bridging characterization is poised to inform the rational design of individual particle geometries and their (templated) assembly within ordered architectures in a manner that is cognizant of the unique interplay of chemo-mechanically coupled electrochemical processes in distinct material systems.

### Particle geometry: constrained topologies

**Fig. 6:**
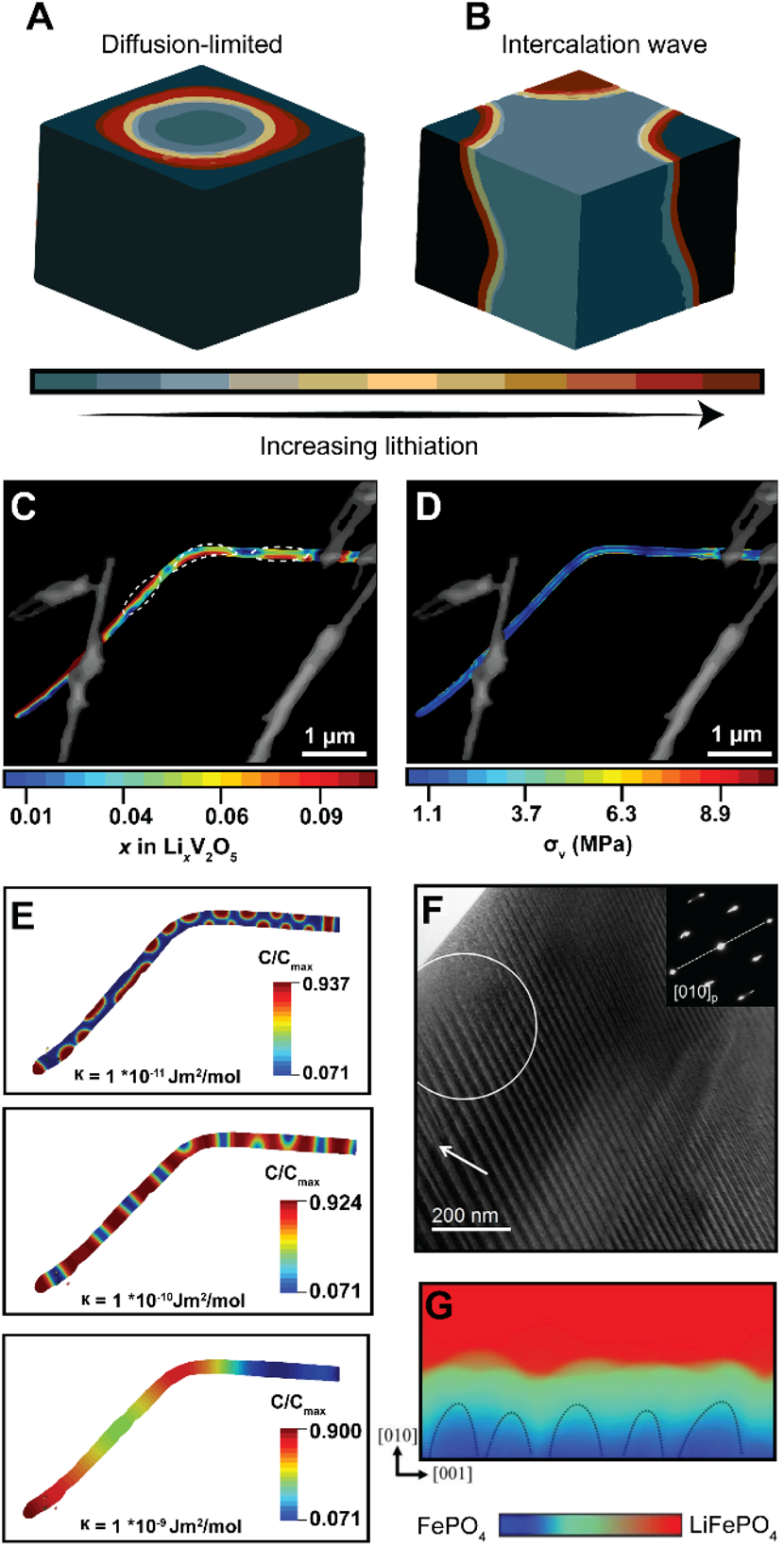
Contrasting patterns of phase multiphasic lithiation in electrode materials. (A) In a diffusion-limited regime, wherein bulk diffusion cannot match the rate of surface reactions, a shrinking-core model is observed. (B) In a nucleation-limited regime, lithiation into a layered material with anisotropic diffusion manifests in intercalation waves stemming from limited nucleation points. (C) Striated phase-separation pattern deduced from STXM measurements of a mechanically bent V_2_O_5_ nanowire. (D) A translation of the lithiation heterogeneities shown in (C) to von Mises stress maps *via* finite element analysis reveals bending-induced control of the resulting stress gradients experienced by the nanowire during delithiation. (E) A complementary phase-field model, modeled after experimental observations, shows the evolution of phase separation patterns as a function of an increasing interfacial parameter, *κ*, which penalizes the formation of sharp phase boundaries (F) shows a stress-driven twinned microstructure in a LMO sample imaged by transmission electron microscopy. (G) Phase-field simulations for LiFePO_4_ show how coherency stress destabilizes a uniform delithiation front and induces filamentary growth patterns. Panels (A) and (B) have been reproduced (adapted) with permission from Fraggedakis *et al.*^98^ Copyright (2020) *Energy Environ. Sci.* (C–E) were reproduced (adapted) with permission from Santos *et al.*,^25^ Copyright (2020) *Mater. Horiz.* (F) has been reproduced (adapted) with permission from Erichsen *et al.*,^102^ Copyright (2020) *ACS Appl. Energy Mater.* (G) has been reproduced (adapted) with permission from Yang *et al.*,^99^ Copyright (2020). *J. Mater. Chem. A*.

In phase-transforming systems, the energetic penalties arising from coherency strain are profoundly modified by the geometry of the active materials (particularly for nanometer-sized particles with large surface stresses) and can often counterbalance the thermodynamic driving forces favoring multiphasic lithiation. Furthermore, the spatiotemporal evolution of phase separation remains closely linked to performance and degradation in LiBs. Phase boundaries directly contribute to differences in local electronic conductivity, reduce interface mobility, and underpin the continuous stress accumulation, which result in the formation of defects, microcracks, and fatigue cracking.^28,97,98^ Thus, the specific patterns of phase separation are closely related to degradation mechanisms. In an attempt to elucidate the conditions that control the morphology of phase separation in electrode particles undergoing intercalation, Fraggedakis *et al.* recently proposed a generalizable scaling law to rationalize distinct phase morphologies.^98^ In a diffusion-limited regime wherein the distribution of intercalated Li^+^ lags behind intercalation at the surface, a “core–shell” morphology is observed (Fig. 6A). Conversely, during a reaction-limited regime, “intercalation waves” originating from a limited number of surface nucleation sites are propagated throughout the volume of the active particle (Fig. 6B). While these simulations capture the importance of balancing surface reactions with diffusion, strain gradients emerging during lithiation, mechanical constraints imposed by geometry, bending, or epitaxial mismatch introduce additional elastic boundary conditions that influence the energy minimizing phase separation pattern,^99^ which creates opportunities to control stress gradients dynamically.

It is important to emphasize the distinction between intercalation-induced strains and strains induced by mechanical constraints in the active material's geometry – the latter is arguably underexplored but nevertheless represents an avenue by which strain can be pre-emptively used to modulate intercalation phenomena. In recent work, we demonstrated how elastic strain introduced prior to (de)lithiation through mechanical bending/clamping profoundly alters phase separation patterns and the resulting stress gradients in individual particles of V_2_O_5_.^25^Fig. 6C shows phase the separation pattern in an individual bent nanowire upon delithiation as imaged by scanning transmission X-ray microscopy (STXM). The real-space distribution of distinctly lithiated phases illustrates the formation of Li-rich domains in the form of regularly spaced striations along the apex of the bend in the nanowire. These observations cannot be adequately explained by a symmetric-core shell lithiation,^49^ a single intercalation wave,^98^ or an asymmetric reaction front propagating on the tensile and compressive sides of particles.^100^ In this work, the system relaxes to adopt a configuration that minimizes the total free energy under applied mechanical constraints. As such, the formation of this distinct lithiation motif is indicative of an equilibrium phase pattern, which for this specific geometry, minimizes the combined penalties from (1) the concentration gradients, (2) coherency strains, (3) and the elastic contributions introduced by the static bend, thereby providing an experimental observation of chemistry–mechanics coupling, at play in a single electrode particle. Strikingly, a translation of experimentally determined lithiation gradients to stress maps *via* finite element analysis^101^ as shown in Fig. 6D suggests that the resulting von Mises stress directly mirrors the altered patterns of phase separation. Here, geometrically imposed strains alter intercalation-induced stresses – illustrating the promise of strain-engineering approach to mitigate deleterious intercalation-induced strains.

To further demonstrate the tunability of phase separation patterns, we employed phase-field modelling to show how phase separation patterns, under the same bending-induced strain, evolve as a function of a gradient energy coefficient, *κ*, which penalizes the growth of sharp interfaces. As shown in Fig. 6E, with an increasing value of *κ*, the apparent geometric effects are decreased, resulting in a phase-separation pattern that is instead dominated by the magnitude of interfacial energy introduced by the gradient energy coefficient. During diffusion, this gradient energy derives from local distortions arising from concentration gradients; during phase transformations, this energy term comprises chemical, mechanical, and even electronic contributions in correlated oxides. Furthermore, it is worth noting that interfacial energy scales with size and is a measure of lattice mismatch across differently lithiated materials, which implies that similar strains could have profoundly different effects in larger particles in this system or active materials with a different lattice relationship between low- and high lithiated phases.^27^

In LiMn_2_O_4_ (ref. 102) and LiFePO_4_,^99^ the strong influence of elastic effects on the phase evolution of single-crystalline particles has been experimentally imaged by transmission electron microscopy (TEM) and modelled by phase-field simulations, as exemplified in Fig. 6F and G, respectively. Together, these results point toward the widely divergent patterns of phase separation accessible within constrained geometries. In a subsequent section focused on electrode geometries, we revisit the use of strain in a practical demonstration to enhance the reversibility of thin-film intercalation electrodes.

### Electrode geometry: epitaxial strains in thin films

The prevalence of intercalation-induced phase transformations in positive electrode materials is both a bane and a boon: on the one hand, these structural changes facilitate continued intercalation, which increases the specific capacity of the electrode material – conversely, phase transformations require considerable energy dissipation, and are the origin of coherency strains preceding fatigue and fracture.^14,51^ In phase-transforming materials such as α-V_2_O_5_, a sufficiently large rearrangement of atomic connectivity renders some transformations irreversible under realistic operating conditions.^14^ Even after imposing constraints on the operating voltage, which inherently affects capacity, reversible and irreversible transformations may lead to a loss of lithium inventory and hindered diffusion.^6,97^ In intercalation batteries, strain is directly coupled to phase transformations through modifications of electronic structure, lattice distortions, and, more generally, modification of relative thermodynamic stabilities across free-energy landscapes. Considering this coupling between chemistry and mechanics, we posit that the less desirable consequences of miscibility gaps might be alleviated to unlock high specific capacities by precisely engineering phase transformations using pre-applied elastic strains.

As introduced in the section on strained-single-particle topologies, the multi-dimensional parameter space of the strain tensor^103^ enables a vast framework wherein the energetics of intercalation reactions in nanostructures with fixed chemical composition can be dynamically tuned through elastic deformations. Strain engineering has been widely implemented in material systems such as semiconductor electronics^104^ and magnetic films^105^ to enhance device performance and has further been used to modulate the d-band positioning and reduce the overpotential of electrocatalytic systems.^106,107^ Despite the promise of strain engineering in battery applications and its prevalence in realistic device structures, only recently have mechanical strains been used to tune the electrochemical properties of electrodes.

**Fig. 7:**
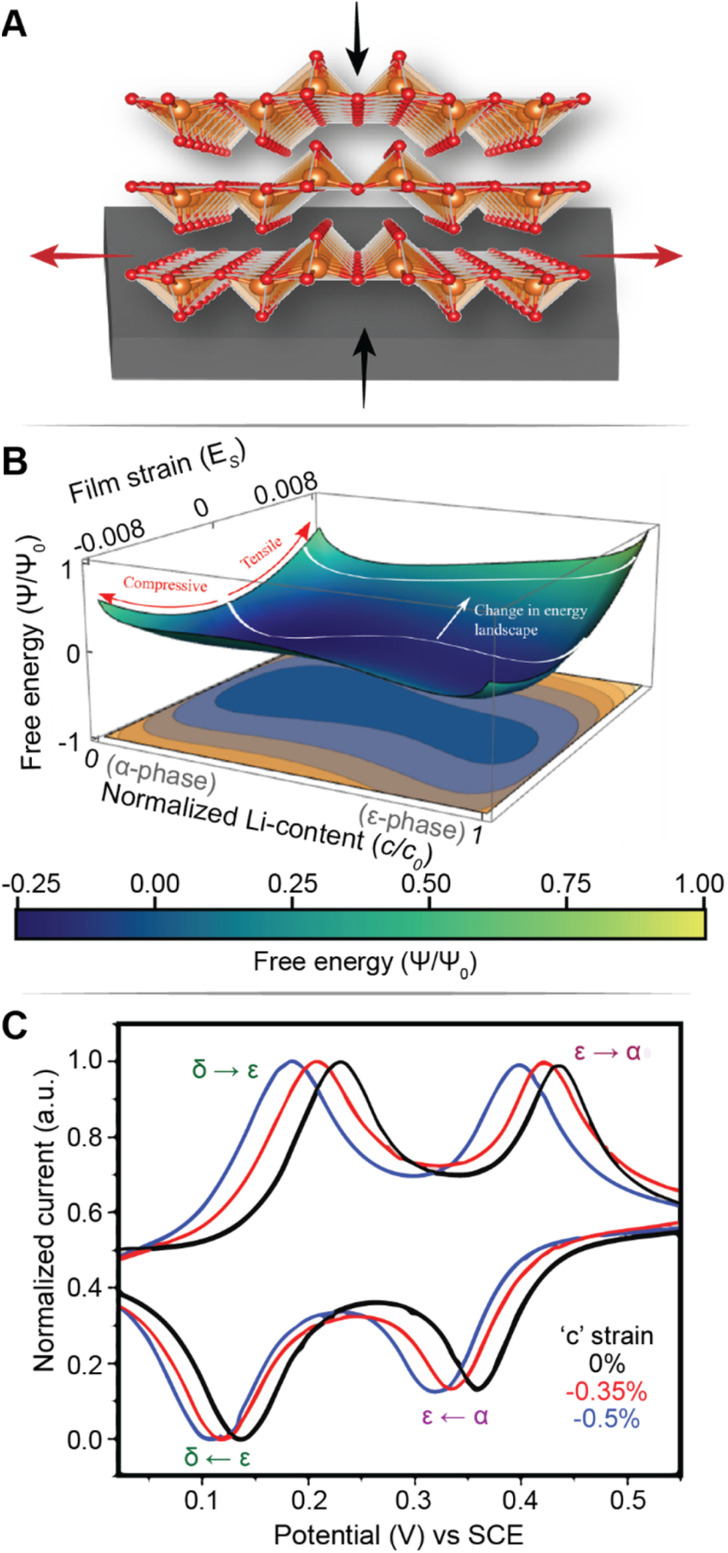
Strain-driven alteration of phase transformations. (A) Illustration of introducing strain in a thin-film cathode material *via* epitaxial mismatch with an incommensurate substrate. (B) 3D plot of the free energy landscape between the α- and ε-phase of V_2_O_5_. The effect of film strain is reflected through a modification of the energy barrier height between the two phases. (B) Has been reprinted (adapted) with permission from Zhang *et al.*,^108^ Copyright (2021) *J. Mech. Phys. Solids*. (C) Cyclic voltammograms of a strained (red and blue), and unstrained (black) V_2_O_5_ thin film, demonstrating a strain-induced shift in intercalation potentials. (C) Has been reprinted (adapted) with permission from Muralidharan *et al.*^34^ Copyright (2017) *ACS Nano*.

Fig. 7A illustrates the basic principle of pre-programming elastic strain through an epitaxial mismatch between a thin-film geometry of the active material and its substrate. Binder-free thin film cathodes have attracted considerable interest for lightweight device applications on account of their ability to eliminate the weight and added complexity of the inactive constituents. One additional benefit to these single-phase (*i.e.*, active material without additives/binders) electrode geometries is the ability to apply varying elastic strains through distinct pairings between the active material and the underlying substrate. In this approach, it is assumed that varying interfacial geometries can lead to distinct properties. For phase-transforming systems, this approach has been leveraged to fundamentally regulate electrochemically induced phase transformations to increase capacity and minimize structural degradation.^108^ In one such example by Zhang^108^ and colleagues, epitaxial strains have been leveraged as a design parameter to modulate the free energy landscape of Li_*x*_V_2_O_5_ and enable a degree of control over its intercalation-induced phase transitions. Fig. 7B shows a 3D plot of the free energy landscape as a function of both Li-composition and epitaxial strains. The effect of epitaxial mismatch is clearly illustrated by this strain-dependent thermodynamic landscape. Notably, with increasing tensile strain, the barrier height between transformations is lowered, effectively mirroring the trends observed from a reduction in particle size. Theoretical calculations predicting a lowering of voltage plateaus with increasing film strain are supported by experiment and are in good agreement with the results from Pint and co-workers, which estimates a modulation of the intercalation potentials by *ca.* 40 mV for applied strains <2% as shown in Fig. 7C.^34^ Interestingly, the most significant shifts in voltage curves are observed for the δ → γ and γ → ω transformations, which are typically irreversible on account of their large structural rearrangement.^14,109^ With only *ca.* 2 GPa of stress, the γ → ω voltage plateau is lowered below 2 V, effectively expanding the operational voltage window and increasing reversibility by mitigating large stresses associated with this transformation. It is worth noting that directional compressive strains can increase the net volume of the intercalation host, enlarge the interlayer spacing and reduce Li^+^ diffusion barriers, thereby also modifying diffusivity.^98^ We revisit this relationship in a later section, discussing the role of pre-intercalation in reducing activation energy barriers for ion transport. While highlighted here for V_2_O_5_, the concept of leveraging strain to modify the energetics of phase transformations can be adapted to several systems – by simply tuning the barrier height between transformations, solid-solution lithiation regimes can be unlocked, and the obstacles presented by phase transformations in otherwise promising positive electrode materials can begin to be addressed.

Currently, most models attempting to capture the role of strain on phase transformations do not account for the asymmetric change in the free energy curves of the two phases possible as a result of strain energy contributions to fundamentally different crystal structures with varying structural anisotropy. In material systems with metal-to-insulator transitions, the application of strain has been shown to alter the relative stability of phases significantly, thus poising systems at the cusp of transitions^110^ – this principle is highlighted in a later section on alloying and site-selective modification strategies. Similarly, precise control of transformation pathways through strain modulation of low- and high-lithiated phases can potentially alleviate stress accumulation and fatigue during cycling. Addressing this knowledge gap will require an improved understanding of the balance between driving forces for multiphasic lithiation and translation to actionable materials design and strain coupling stratagems.

### Electrode geometry: from interconnected particle networks to the rational design of 3D porous electrode architectures

**Fig. 8:**
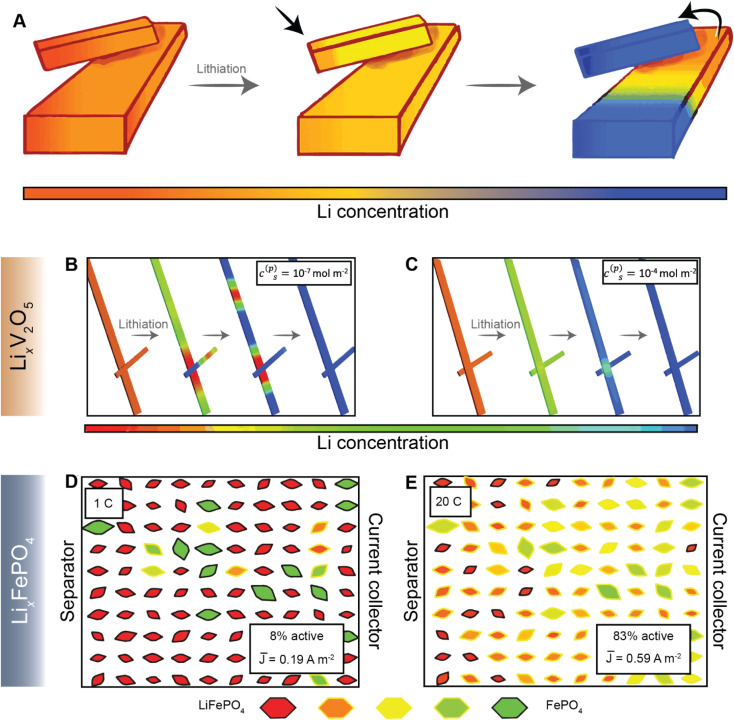
Lithiation across interconnected particles and reaction rate-induced changes in phase separation patterns. (A) Schematic depiction of lithiation across interconnected particles. The smaller particle becomes preferentially lithiated due to a higher surface-area-to-volume ratio whilst depleting the larger interconnected particle. (B) and (C) show the evolution of average lithium concentration in a pair of interconnected particles during comparably slow (10^−7^ mol m^−2^) and fast (10^−4^ mol m^−2^) reaction rates (*c*^(p)^_s_), respectively, on the electrode/electrolyte interface. Here, the rapid reaction suppresses phase separation. A similar current-induced transition from particle-by-particle to concurrent (de)lithiation is observed for LiFePO_4_, when the discharge rate is changed from 1C (D) to 20C (E). Portions of panel (A) have been reproduced (adapted) with permission from de Jesus *et al.*,^114^ Copyright (2017) *J. Mater. Chem. A*. Panels (B) and (C) have been reproduced (adapted) with permission from Zhao *et al.*,^41^ Copyright (2017) *RSC Adv.* (D) and (E) have been reproduced (adapted) with permission from Li *et al.*,^116^ Copyright (2014) *Nat. Mater.*

Most current commercial positive electrode materials are manufactured *via* the preparation of a slurry comprising active material, binder, solvent, and conductive additives.^111^ In addition to balancing average porosity and tortuosity to maximize energy and power density, geometric irregularities should be monitored to avoid large heterogeneities in local potentials.^112,113^ In recent work, we have examined interconnected V_2_O_5_ particle networks and observed that in a reaction-limited mode, nucleation and growth of a Li-rich particle proceeds with depletion of Li-ions from a connected adjacent particle (Fig. 8A).^41,114^ The highly lithiated phase propagates as an intercalation wave across a single nanowire in a “winner takes all” fashion instead of concurrent lithiation across the interconnected network. Orvananos *et al.*, have examined phase separation across networks of particles and devised a two-particle model; as per this model, at a constant applied current, the particle closer to the separator undergoes rapid lithiation whilst depleting a particle that is further away from the separator.^115^ The operation of particle-by-particle lithiation and the specifics of network inhomogeneities are dependent on the trade-offs between interfacial barriers for Li-ion migration, surface reaction rates, and barrier to nucleation of the Li-rich phase. Furthermore, the spatial orientation of individual particles in the agglomerate represents an important geometric consideration. Phase-field simulations further reveal that rapid reaction rates can mitigate phase separation, enabling single-phase solid-solution lithiation across an individual particle as well as a network of particles, as shown in Fig. 8B and C, for Li_*x*_V_2_O_5_ (ref. 41), and Fig. 8D and E for LiFePO_4_ (ref. 115). These results point to the imperative to minimize dimensional variations in electrodes comprising phase-separating materials and to ensure a high degree of interconnectedness to alleviate intra- and inter-particle phase separation.^41,114^

The maturation of additive manufacturing, in conjunction with templating methodologies, has enabled the possibility of fabricating electrodes with precise and often complex geometries. By leveraging the interplay between geometry and electrochemistry to deterministically define diffusion pathways, charge and mass transport can be directed across length scales. In pursuit of greater control over electrode porosity, tortuosity, and three-dimensional (3D) microstructure, several methods of engineering thick electrodes for high-performance batteries have been developed. These broadly include subtractive designs based on sacrificial templates and additive manufacturing designs using 3D printing.^117–119^ Several hybrid heterostructured designs have been examined that interface active positive electrode materials with conductive materials such as lamellar stacks of 2D materials and graphene to enable high-rate performance.^120,121^

**Fig. 9:**
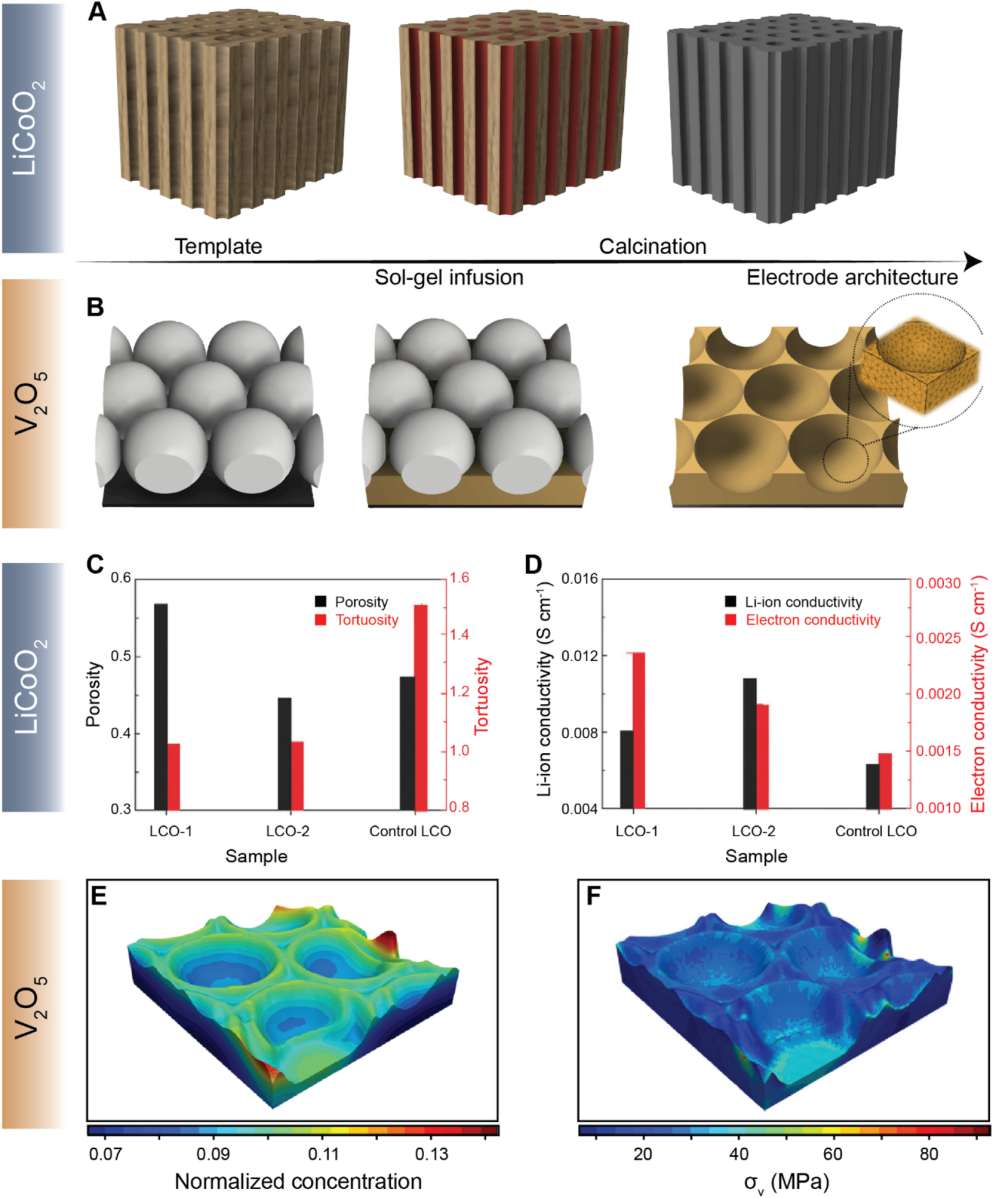
Leveraging geometry to improve cathode performance. A schematic depiction of a sol–gel process utilized to fabricate a templated LCO cathode using a wood template and a V_2_O_5_ porous architecture using a colloidal crystal template is shown in (A) and (B) respectively. A comparison of tortuosity and porosity of the templated LCO-1, templated LCO-2, and control LCO architectures is shown in (C). (D) Comparison of Li-ion and electron conductivities between the templated LCO-1 and LCO-2 cathodes against the control LCO. The normalized equilibrium concentration map shown in (E) shows that lithium redistributes homogenously across the continuously curved V_2_O_5_ 3D architecture. (F) Stress map of the Li_*x*_V_2_O_5_ inverse opal structure derived from a finite element simulation based on the spatial lithiation pattern as imaged by X-ray spectromicroscopy. Panels (C) and (D) have been reprinted (adapted) with permission from Lu *et al.*,^128^ Copyright (2018) *Adv. Mater.* Panels (E) and (F) have been reprinted (adapted) with permission from Andrews *et al.*,^129^ Copyright (2020) *Matter*.

Navigating the vast parameter space at the intersection of chemistry and geometry is nevertheless a complex task. While high-energy density can be achieved by increasing electrode thickness, for example, extended Li-ion transport pathways can dramatically increase the resistance for diffusion, stymying high-rate performance.^122^ To this end, several methods of engineering electrode structures have been explored^117^ to control the structural factors of electrodes, as demonstrated by the recent collection of low-tortuosity electrodes spanning multiple cathode chemistries.^42,43,123–125^ X-ray and electron tomography have emerged as vital tools for mapping the 3D spatial distribution of active materials and the effective tortuosity^126^ and tortuosity anisotropy, which is a function of crystallite geometry and processing parameters.^124^ Chiang and co-workers introduced the idea of dual pore porosity to reduce tortuosity of porous electrode architectures.^43^ These authors used a combination of aligned channels with a porous matrix, controlling, yielding improved rate performance.^127^ For instance, Yu^127^ and co-workers have demonstrated a gradient electrode design characterized by porous channels that have differently sized openings on either end. Such a gradient design allows for precise modulation of kinetics of high-energy-density structures.^127^ Yu and co-workers have further designed a low-tortuosity high power/energy density LiFePO_4_ electrode with substantial active material loading and utilization by fabricating a 3D network for facile ion and electronic transport using templated phase inversion.^125^ In this perspective, templated LiCoO_2_ (LCO) and V_2_O_5_ architectures are highlighted in Fig. 9A and B, respectively. Taking inspiration from nature, Lu and colleagues fabricated a LCO architecture *via* a sol–gel process (Fig. 9A) that parallels the water transport channels found in wood.^128^ The built-in microchannels in the positive electrode structure significantly reduce tortuosity and improve Li-ion conductivity by *ca.* two-fold, as shown in Fig. 9C and D, respectively. In our recent work, we employed a similar sol–gel method to fashion a monolayered inverse opal structure from a colloidal crystal template, as shown in Fig. 9B.^129^ Incorporating continuous curvature accelerates Li-ion diffusion kinetics whilst retaining homogeneity during lithiation. In addition to the ability of the patterned electrode to overcome multiphase coexistence (Fig. 9E), this electrode architecture enables stress relaxation (Fig. 9F) during lithiation, which presents a substantial advantage over planar thin-film electrodes, enabling considerably improved mechanical resilience and improved rate performance upon extended cycling.^130^ The latter work on V_2_O_5,_ in particular, demonstrates how electrode topology can be used not only to maximize energy density but also to optimize the macroporous structure to mitigate the coupled effects of lithiation heterogeneities and stress.

**Fig. 10:**
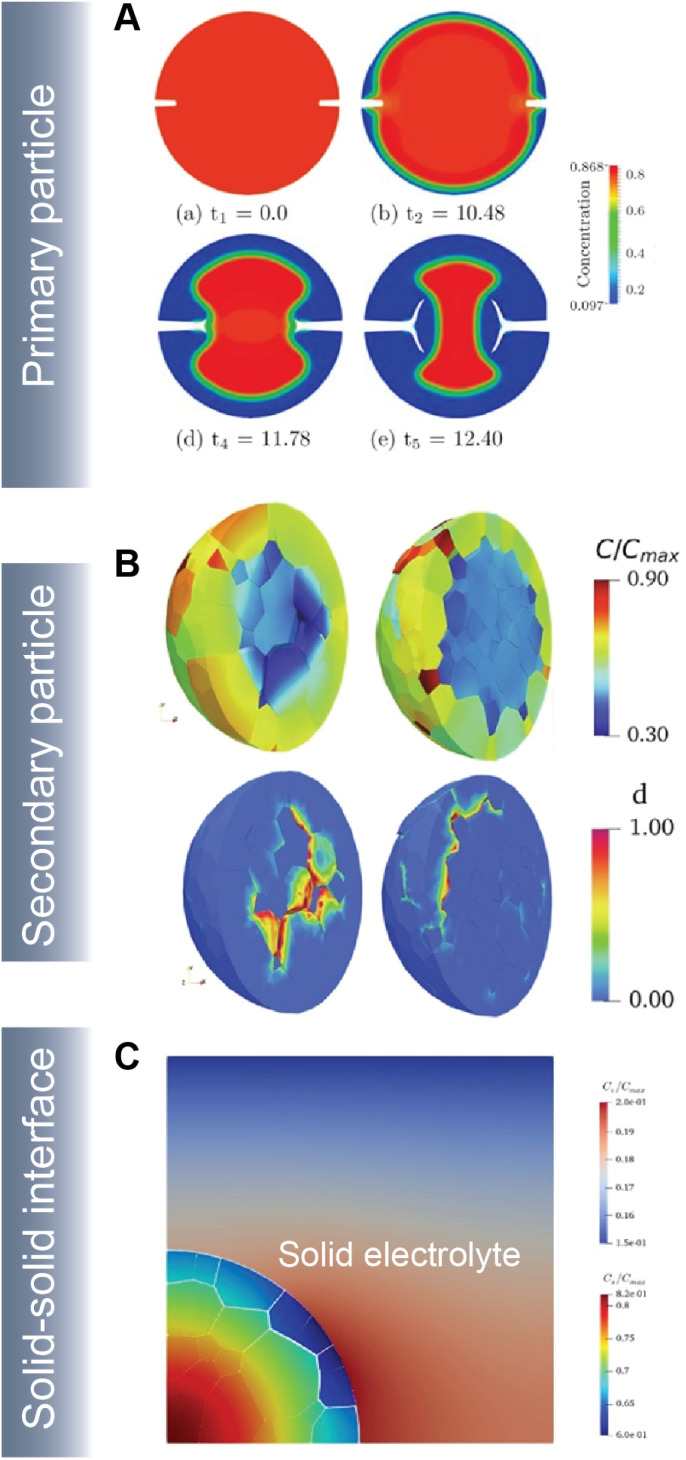
Simulating crack propagation in active electrode materials. (A) Evolution of fracture and phase segregation during delithiation of a cylindrical particle. Reprinted (adapted) with permission from Xu *et al.*^45^ Copyright (2016) *GAMM-Mitteilungen* (B) lithium concentration and damage distribution in NMC particles with different numbers of primary particles. Reprinted (adapted) with permission from Bai *et al.*^181^ Copyright (2021) *Int. J. Solids Struct.* (C) Concentration profile and damage parameters for a composite cathode system in an ASSB. Reprinted (adapted) with permission from Rezaei *et al.*^20^ Copyright (2021) *J. Mech. Phys. Solids*.

As part of a dynamic mechano-electrochemical system, electrodes are continuously subjected to electrochemically-driven stimuli such as mechanical deformation and thermal gradients. In intercalation cathodes, fracture results from repetitive deformation and originates from the accumulation of dislocations and inhomogeneous swelling and shrinkage during (dis)charge. Crack initiation can proceed at primary (Fig. 10A) and secondary (Fig. 10B) particle levels and often hinders ionic and electronic transport accelerating resistance rise and capacity face. In ASSBs, the solid–solid interface between the active material and solid-electrolyte gives rise to physical constraints that cause interfacial delamination during the swelling and shrinkage of the active particle during de(lithiation) (Fig. 10C).

Recently, it has been shown that the rational design of 3D electrode geometries opens new opportunities to incorporate stress relief mechanisms *via* prescribed buckling mechanisms during lithiation.^118,131^ Notably, the bulk of these observations pertain to anode materials, which, while they are subject to large volumetric expansion upon lithiation, are also much more ductile, enabling stimulus-responsive shape memory behavior. An incomplete understanding of the interplay between geometry and the thermodynamics of phase transformations represents a critical bottleneck for similar targeted designs of deformation mechanisms in smart, stimulus-responsive positive electrodes, which tend to be much more brittle. Despite the apparent challenges, incorporating dynamic deformations through electrode architectures, such as programmable slippage in layered materials or morphogenetic coupling of phase separation patterns to reactions at the cathode/electrolyte interface, could enable active materials to transform dynamically and reversibly in response to local modulations of lithium concentration, thereby sensing and counteracting damage. As a potential example of biomimetic morphogenesis,^132^ a closed-loop reaction sequence can be envisioned in which phase-separating microstructures formed in positive electrodes during lithiation drive local thermal gradients and surface reactions, which in turn catalyzes the formation of a cathode/electrolyte interface. The dynamical precipitation, redissolution, and evolution of the CEI can provide a means to maintain a robust electrode/electrolyte interface, including in ASSBs.

## 4. Tuning chemistry–mechanics coupling through atomistic materials design

A viable insertion host must satisfy several fundamental requirements. An abundance of interstitial sites and accessible redox centers are necessary to realize fundamental intercalation and redox functions, whereas low-site-to-site diffusion barriers and facile solvation/desolvation at interfaces are critical to unimpeded diffusion and interfacial processes. Additionally, cell performance hinges on the compatibility between the cathode, the anode, and the electrolyte. A large difference in chemical potential between electrodes is preferred to maximize open circuit potential, but not to the extent that the thermodynamically uphill process is blocked. Furthermore, a growing emphasis on levelized costs and sustainability places additional crustal abundance and supply chain constraints on the search for next-generation electrode materials.^133^ In this regard, discovering altogether new battery chemistries that outperform existing materials remains a grand challenge for energy storage. We discuss some aspects of materials design, specifically pre-intercalation, polymorphism, and alloying, as a means of modifying the atomistic and electronic structure, and thus phonon dispersion, which thereby affords considerable control over electrochemistry–mechanics coupling.

### Primed for success: pre-intercalation of electrode materials

Conceptually, pre-intercalation strategies involve the insertion of small fractions of an electrochemically inactive or semi-active guest species into the host structure before operation.^134^ Intercalants can be inserted through chemical (during synthesis or through post-synthetic topochemical insertion) or electrochemical methods and typically include organic and inorganic ions, solvents, or organic molecules.^135,136^ Pre-intercalation strategies for electrode materials have received increasing attention on account of the often-synergistic effects between the guest species and the transport properties of the intercalation host.^137–139^ Pre-intercalated species can have “pillaring” or “propping” effects, modulate local dielectric environments, and solvate cations within confined galleries.

**Fig. 11:**
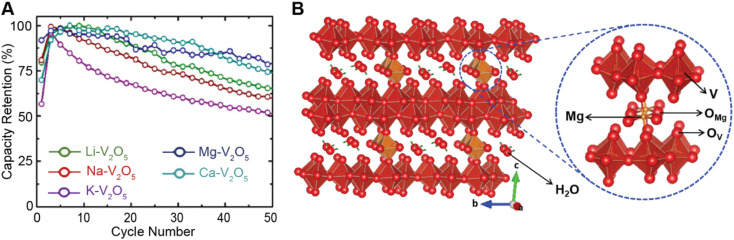
Improved electrochemical performance of pre-intercalated electrodes. (A) Capacity retention from bilayered δ-M_*x*_V_2_O_5_ (M = Li, Na, K, Mg, Ca) electrode materials. Reproduced (adapted) with permission from Clites *et al.*,^136^ Copyright (2018) *Energy Storage Mater.* (B) Schematic illustration of Mg_0.3_V_2_O_5_·1.1H_2_O. Here, pre-intercalated Mg^2+^ reinforces the layered structure, whereas the lattice water solvates Mg-ions and enables fast cation diffusion. Reproduced (adapted) with permission from Xu *et al.*,^182^ Copyright (2019) *Chem*.

In layered materials, the most well-recognized mechanism of pre-intercalation is the cooperative effect between intercalant-induced interlayer expansion and diffusivity, specifically the role of the intercalated species in reducing activation energy for site-to-site ion migration. As discussed in Section 2 the insertion of guest species leads to local distortions and volumetric expansion driven primarily by electrostatic repulsions. In turn, it has been shown that the activation barrier for Li^+^ hopping is strongly correlated to the local electrostatic interactions and Li slab distance.^140^ Generally, pre-intercalation expands the interlayer spacing, relieving electrostatic repulsion between neighboring Li-ions, thereby facilitating diffusion. Recent work by Pomerantseva and co-workers demonstrates the enhanced ionic diffusion resulting from the application of chemical pre-intercalation to prepare a series of bilayered δ-M_*x*_V_2_O_5_ (M = Li, Na, K, Mg, Ca) positive electrode materials.^136^ Their findings demonstrate the efficacy of systematically tuning the interlayer spacing to facilitate diffusion by varying the size and charge of the pre-inserted ions. Here, the Mg-stabilized δ-V_2_O_5_ bronze, which has the largest interlayer spacing, showed the highest capacity retention rate, as demonstrated in Fig. 11A. In addition to facile diffusion derived from increased interlayer spacing, the improved cyclic stability from multivalent pre-intercalation is rationalized based on the enhanced interactions between the pre-intercalant and the host, which stabilize the layered structure. This so-called “pillaring effect” has been recorded during investigations into the bilayered Mg_0.3_V_2_O_5_·1.1H_2_O structure shown in Fig. 11B. Upon electrochemical magnesiation, the pre-inserted Mg^2+^ acts as a structural element, connecting adjacent layers that diminish stress-inducing distortions during cycling.^139^ and the incorporated lattice water effectively enables the mobility of polarizing Mg^2+^-ions, thereby yielding excellent electrochemical performance. During subsequent demagnesiation, the crystal structure of the Mg_0.3_V_2_O_5_·1.1H_2_O is restored, thereby demonstrating good structural reversibility.

While pre-intercalation generally facilities diffusion and alleviates large volume changes, thereby minimizing stress accumulation, it comes at a price. Pre-intercalation inevitably occupies a fraction of the interstitial sites that would otherwise be available for Li^+^ during (dis)charge. Furthermore, in some pre-intercalation electrodes, the pre-intercalated species might become deintercalated from the host structure, reversing their advantages and possibly giving rise to unwanted side reactions.^134,141^ Nevertheless, pre-intercalation strategies provide a readily accessible approach to minimizing stress gradients, especially for “beyond-lithium” technologies.

An additional advantage of pre-intercalation accrues from the formation of mixed-valence states (*i.e.*, V^5+^ and V^4+^), which results in improved electronic conductivity.^142^ In the case of V_2_O_5_, partial occupancy of narrow V 3d_*xy*_-derived split-off conduction band states as a result of pre-intercalation alleviates a major polaronic bottleneck to Li-ion diffusion.^143^ Furthermore, the structural modifications resulting from the pre-incorporation of guest species necessarily alter the energetics of phase transformations and the lattice relationships between low- and high-lithiated phases, as discussed in a later section on doping/alloying.

### Accessing altered intercalation mechanisms and chemistry–mechanics coupling in metastable phase space

The known crystal structures of periodic crystalline solids often reflect the minimum energy structural configuration accessible for a specific combination of atoms. Nevertheless, alternative structural arrangements with vastly different atomic connectivity and altered lattice structure can be stabilized under specific constraints, spanning changes in temperature, pressure, alloying, and defects, and exhibit starkly different properties.^144^ These alternative “metastable” polymorphs with reconfigured atomic connectivity exhibit entirely distinctive phonon dispersion and Li-ion diffusion pathways for the same exact composition. As such, these materials are intriguing model systems to develop an intuition for coupling of chemistry–mechanics coupling and are furthermore of enormous technological importance because they afford the ability to access well-defined Li-ion diffusion pathways with precisely tunable migration barriers.^145,146^ The concept of metastability finds particular resonance in intercalation batteries which often operate far from equilibrium. For example, in the canonical LiFePO_4_ system, the spinodal region between the delithiated and lithiated phases (*i.e.*, FePO_4_ and LiFePO_4_) progressively vanishes with an increasing current rate.^147,148^ For particles below a critical size, a metastable continuous solid solution is favored, bridging the two-end member phases. In the case of MnO_2_, the presence of metastability is demonstrated by the broad range of Mn–O connectivity encompassing numerous polymorphs^149^ with diverse electrochemical properties.^150^

**Fig. 12:**
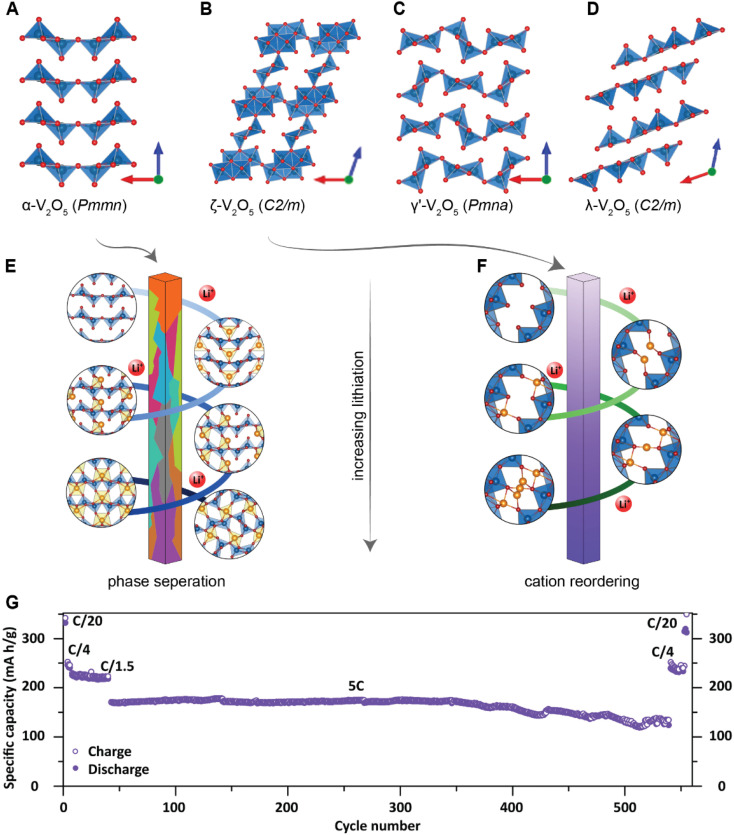
Contrasting intercalation phenomena in V_2_O_5_ polymorphs. (A) Orthorhombic α-V_2_O_5_, represents the thermodynamic sink of the vanadium oxide phase diagram. (B–D) show the structures for the metastable ζ-V_2_O_5_, γ′-V_2_O_5_, and λ-V_2_O_5_ polymorphs, respectively, which have been stabilized by topochemical de-intercalation of native cations from ternary vanadium oxide M_*x*_V_2_O_5_ bronzes. In α-V_2_O_5_, increasing lithiation brings about distortive structural transformations, which often coexist at the single-particle level as shown in (E). In sharp contrast, ζ-V_2_O_5_ accommodates homogenous lithiation as shown in (F) through Li-ion reordering along the 1D tunnels. By alleviating distortive phase transformations, ζ-V_2_O_5_ shows excellent capacity retention as shown in (G). (E–G) were reproduced (adapted) with permission from Luo *et al.*,^155^ Copyright (2022) *Proc. Natl. Acad. Sci.*

In past work, we have explored the use of several metastable V–O frameworks (Fig. 12B–D) stabilized by topochemical extraction of cations from ternary M_*x*_V_2_O_5_ bronzes as potential electrodes of Li^+^ and multivalent batteries.^144,151–154^ Many of these structures exhibit distinctive vanadium–oxygen connectivity and stacking of [VO]_5_ square pyramids and [VO_6_] octahedra, thus defining characteristic interstitial sites along which Li-ions can migrate. The diffusion pathways accessible in such structures are substantially different from the thermodynamically stable phase of V_2_O_5_ shown in Fig. 12A. As such, they afford entirely distinctive magnitudes and directionality of coupling to strain fields.

As previously shown in Fig. 4, the insertion of lithium into α-V_2_O_5_, gives rise to several distortive phase transformations that contribute to its limited rate performance and give rise to phase inhomogeneities that eventually result in degradation.^14,25^ Despite a high theoretical capacity (443 mA h g^−1^), the consequences of phase separation combined with intrinsically low conductivity continue to plague the thermodynamic sink of the binary V–O phase diagram. In a direct comparison between α-V_2_O_5_ and the metastable one-dimensional (1D) tunnel-structured ζ-V_2_O_5_ polymorph, shown in Fig. 12B, we demonstrate how variations in structural and electronic motifs of the two polymorphs provide access to profoundly different intercalation mechanisms as schematically depicted in Fig. 12E and F for α-V_2_O_5_ and ζ-V_2_O_5_, respectively. In sharp contrast to α-V_2_O_5_, wherein intercalation induces several phase transformations, a combination of “frustrated coordination environments” and a greater degree of covalency in ζ-V_2_O_5_ enables homogenous intercalation through cation-reordering along the tunnels.^152,155^*Operando* MOSS measurements performed during electrochemical cycling reveal consequentially less stress accumulation in the metastable polymorph as compared to its thermodynamic counterpart, which is furthermore consistent with its more rigid 1D tunnel framework. The excellent cycling stability exhibited by ζ-V_2_O_5_ is shown in Fig. 12G, which captures the implications of an entirely distinctive insertion and diffusion mechanism.^155^

As an additional point of comparison, the open structure of the metastable γ′-V_2_O_5_ intercalation host shown in Fig. 12C accommodates Li-ion intercalation through an altogether different mechanism from α-V_2_O_5_ and ζ-V_2_O_5_.^154–156^ High-resolution single-crystal-to-single-crystal transformations of γ′-V_2_O_5_ reveal the presence of stacked layers with an up–down–up–down motif of VO_5_ square pyramids that reversibly fold as a function of lithiation.^152^ This structural flexibility has already shown considerable promise for beyond-lithium applications involving the insertion of larger cations^154,156^ or multivalent species that may exacerbate diffusion impediments owing to their strong polarizing nature.^151,153^

Metastability as a design principle is fundamentally rooted in the thesis that atomistic and electronic structure can be controlled independently from composition. At present, the promise offered by altered structure–property relations in metastable compounds remains underexplored as a result of fundamental knowledge gaps regarding the accessible metastable phase space. An expanded synthetic toolset combined with a greater understanding of energy landscapes holds promise for atomistic design to finely modulate strain coupling. The implementation of geometric design ideas outlined in Section 3 to metastable polymorphs holds promise for accessing finely engineered systems that encode both atomistic and mesoscale design characteristics for high accessible capacity and mechanical resilience upon prolonged cycling.

### Modulating lattice relationships between low- and high-lithiated phases through alloying

**Fig. 13:**
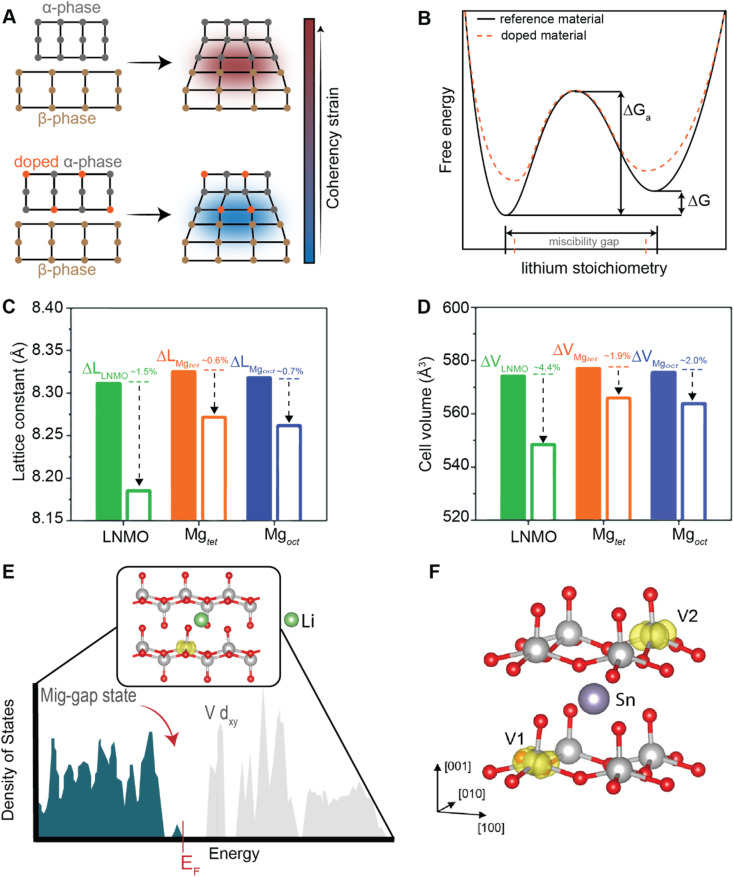
Electronic and atomistic structure origins of dopant-induced modifications in electrode materials. (A) Schematic illustration of the formation of strained phase boundary between two incommensurate phases. Here, substitutional doping lowers the elastic misfit, therefore reducing the coherency strain between the two phases upon phase boundary formation. Doping strategies fundamentally modify the thermodynamic landscape of the system in several ways. (B) Schematically depicts a change in the relative stability of two phases (Δ*G*), a modification to the activation energy barrier for the phase transformation (Δ*G*_a_) and narrowing of the miscibility gap. (C) and (D) Contrast the changes in lattice constants and cell volumes, respectively, during delithiation in the biphasic regime of undoped and Mg-doped LNMO. Mg-doping in LNMO reduces the elastic misfit between the low- and high-lithiated phases. (E) Shows the density of states in α-V_2_O_5_ – like several electrodes based on transition metal oxides, the combination of narrow 3d bands and strong electron correlation engenders the formation of polarons as depicted in the upper panel. The coupling of polarons with nearby Li-ions introduces additional diffusion barriers. (F) Sn-doping into V_2_O_5_ stabilizes two polarons at different V centers. The stabilization of polaronic states before lithiation destabilizes additional polaron formation from the subsequently inserted Li-ion. (C) and (D) Were reproduced (adapted) with permission from Kang *et al.*,^167^ Copyright (2021) *J. Mater. Chem. A*. (F) was reproduced (adapted) with permission from Suthirakun *et al.*,^176^ Copyright (2018) *J. Phys. Chem. C.*

Despite adequate electrochemical performance from the early adapted^157^ LiCoO_2_ (LCO), high cost and mediocre capacity quickly gave rise to alternatives such as LiNiO_2_ (LNO) which delivered much higher capacity at lower prices.^158,159^ However, cathodes comprising pure LiNiO_2_ suffer from low cycling stability, which leads to capacity fade rendering them unsuitable for commercial applications. Today, some of the most widely used cathodes in commercial batteries result from a mixture of LCO and LNO supplemented with various dopants in the form of Li[Ni_*x*_Co_*y*_M_*z*_]O_2_ (M = dopant such as Al).^160^ Presently, doping is a well-recognized strategy to improve the performance of electrode materials. Broadly, the documented benefits of doped electrode materials include: (1) the formation of mixed valency, which typically increases conductivity;^161^ (2) incorporation of redox inactive species to expand the interlayer spacing;^162^ (3) the stabilization of defects, which enable faster Li-ion diffusion and can nucleate phase transformations;^163^ (4) a decrease in charge-transfer resistance, which facilities faradaic reactions;^164^ (5) additional control over particle morphology;^165^ the importance of which has been highlighted in this work; and (6) crystallographic design of structural transformation pathways^71,79^ to preserve the crystal structure (Fig. 13A). Arguably, the individual electronic and structural contributions, from doping to the kinetics and thermodynamics of phase transformations, are less understood. This section aims to provide a perspective on the importance of engineering phase transformations through doping/alloying with the help of recent observations in this field.

Recent advances in understanding crystallographic transformation pathways and elastic misfits have been leveraged to engineer phase transformations, for example, by fundamentally altering the energetic barriers and crystallographic misfits between phases through the incorporation of dopants, as shown in Fig. 13B. The origin of enhanced electrochemical performance of substituted LiFePO_4_, especially at high current densities, for example, has been rationalized based on a decrease in the lattice mismatch between the lithium-rich and lithium-poor phases.^31,166^ Research efforts led by Whittingham and co-workers showed that at room temperature, both Li_0.5_FePO_4_ and Li_0.5_Fe_0.85_V_0.1_PO_4_ exist as two phases within the miscibility gap, indicating preservation of the double-well potential for the V-substituted electrode material, which favors phase separation at equilibrium. During lithiation, however, a single-phase-like behavior is observed for the V-substituted end members indicating operation of a nonequilibrium mechanism of lithium incorporation, which bypasses nucleation and growth of a second phase, can be kinetically accessed as a result of reduced lattice misfit, as illustrated in Fig. 13A, and a change in the free energy landscape, as shown in Fig. 13B.

Kang and colleagues recently unraveled a similar mechanism in Mg-doped LiNi_0.5_Mn_1.5_O_4_ (LNMO) using first-principles calculations.^167^Fig. 13C and D show the effects of Mg doping at either tetrahedral (Mg_tet_) or octahedral sites (Mg_oct_) of the LNMO structure. Based on the relative crystal misfit between the lithiated and delithiated states, the average lattice misfit for the unsubstituted LNMO was estimated to be 1.5% which stands in stark comparison to the *ca.* 0.6% and 0.7% misfits calculated for Mg_tet_-LNMO and Mg_oct_-LNMO, respectively (Fig. 13C). In addition to stabilizing an extended solid-solution regime during the later stages of delithiation in Mg-LNMO, a reduced phase transformation barrier in the biphasic region of the Mg-substituted cathode reveals an apparent dopant-induced change to the thermodynamics of the two-phase reaction. Furthermore, the volumetric changes undergone by LNMO are *ca.* two-fold greater than its Mg-substituted counterparts (Fig. 13D). Analogous to the pillar effects discussed for pre-intercalation, this doping strategy stabilizes the Mg-LNMO structure, further increasing structural stability. A similar stabilization effect has been utilized to mitigate the intrinsic structural instability of LNO, resulting from internal stress caused by the repetitive H2 ⇆ H3 phase transitions through W-doping. At only 2 mol% W, W-LNO shows a 21.8% increase in capacity retention after 100 cycles.^168^

Related studies in phase-transforming materials with shape memory,^169^ electrocaloric,^170^ and memristive properties,^171^ which closely resemble phase transformation characteristics in layered cathodes, showcase the versatility of this design principle which essentially aims to minimize the unwanted consequences of distortive transformations by tuning composition and lattice compatibility. Based on the nonlinear theory of Martensitic transitions, geometric compatibility between transformed and parent phases dominates hysteresis width and phase coexistence regimes.^172^ Selecting dopants that diminish symmetry variations across the intercalation-induced phase transformation by modulating the middle eigenvalue and co-factors of the stretch tensor of the transformation hold promise for alleviating the stresses accumulated as a result of distortive transformations. In an exemplary case from Chluba *et al.*, shape memory alloy films based on TiNiCu, which typically experience fatigue after only a few cycles, were able to be reversibly cycled over 10 million times following careful crystallographic engineering.^169^ Drawing on insights from shape-memory alloys, Zhang and Balakrishna recently established several microstructural design principles to stabilize phase transformation microstructures in intercalation materials with minimum volume changes and stress-free interfaces.^79^

In intercalation systems, electronic structure plays a role in mediating the energetics of Li-ion diffusion.^173,174^ In the canonical α-V_2_O_5_ cathode, for example, the presence of a narrow split-off conduction band derived from V 3d_*xy*_ states results in the stabilization of small polarons, as depicted in Fig. 13E, which brings about atomic-scale traffic jams that amplify lithiation gradients.^143^ In addition to the anticipated structural distortions induced by doping, which are intrinsically coupled to electronic structure *via* perturbation of band overlap, the incorporation of dopants introduces impurity levels that could increase carrier density and fundamentally alter transport properties. Such an approach with extended reversible solid-solution lithiation has been recently observed for Mo-doped V_2_O_5_ wherein Mo alloying induces a pre-transformation to a distorted phase that is similar in structure to the lithiated phase.^175^ Conceptually, the potential efficacy of improving electrochemical performance by tuning electronic structure has also been demonstrated by the tunnel-structured ζ-V_2_O_5_ polymorph.^14,153^ Compared to α-V_2_O_5_, ζ-V_2_O_5_ has broader, more degenerate d-bands, which gives rise to a lower effective mass and a resulting increase in the mobility of the coupled polaron diffusion phenomena. DFT+U calculations performed on the Sn-doped V_2_O_5_ system demonstrate similar promise.^176^ As shown in Fig. 13F, interstitial doping of Sn into V_2_O_5_ creates two polarons at different vanadium centers. The pre-existing presence of a polaron between two layers subsequently destabilizes the formation of a smaller polaron originating from the inserted Li^+^. In addition to gap states resulting from localized electron density on VO_5_ units, electronic structure calculations on Sn–V_2_O_5_ suggest that Sn insertion increases the number of charge carriers, improving conductivity. While V_2_O_5_ is a prominent example of diffusion constraints manifesting from electron localization at transition metal centers, the characteristically narrow d-bands and presence of electron correlation effects in many early first-row transition metals enable a generalization of the potentially beneficial aspects of destabilizing polaron formation^177,178^ and thus improving Li-ion diffusion.^179^

**Fig. 14:**
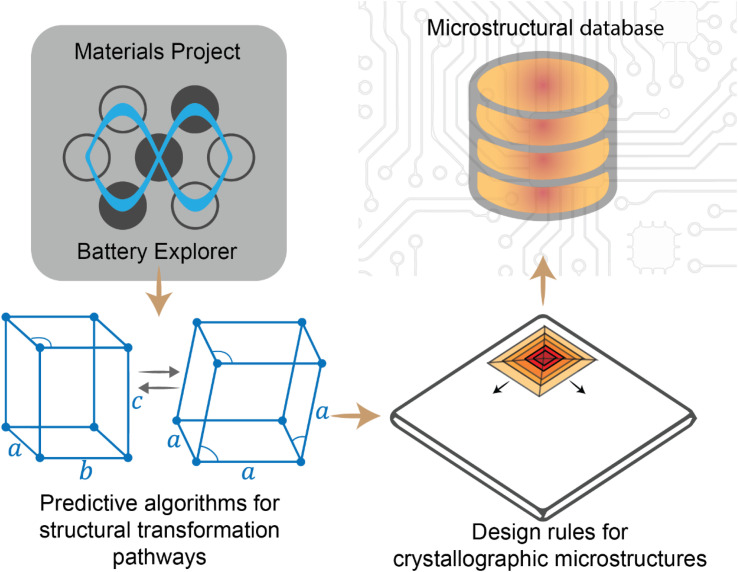
A flowchart of the theoretical framework used to analyze and design structural transformation pathways in intercalation compounds. This framework has been applied to an open-source database (Materials Project) with over *n* > 5000 pairs of intercalation compounds.^71,79^

While the synergistic effects between layered cathode materials and dopants are becoming better understood, one fundamental obstacle to the rational design of doped intercalation cathodes is establishing a causal understanding of the relationship between dopants and the performance of electrode materials. Recent developments in high-throughput computing coupled with expanded access to materials databases offer considerable promise as a means of guiding experimental synthesis beyond Edisonian trial-and-error methods. In recent work, Zhang and Balakrishna^79^ quantified lattice transformation pathways in intercalation compounds (*n* > 5000 pairs of compounds from an open-source structural database) and subsequently identified crystallographic design rules necessary to reduce lattice misfit strains and volume changes during phase transformations. This framework, shown in Fig. 14, has been successfully leveraged to uncover the mechanism for cubic-to-tetragonal structural transformations in Li_1_–_2_Mn_2_O_4_ (ref. 56) and demonstrates the considerable promise of developing a detailed understanding of individual transformation pathways and the availability of control knobs for calibrating structural transformations through alloying.

## 5. Conclusions and outlook

The current state of Li-ion batteries requires substantial breakthroughs to viably approach theoretical capacities at high rates in order to meet increasingly stringent requirements of electric vehicles and grid-level storage. The sparse selection of positive electrodes that satisfy the constraints governing commercial viability is a major stumbling block to electric mobility and decarbonization of the energy grid. Continued progress in electrochemical energy storage at the scale of Terrawatt hours requires a fundamental mechanistic understanding of the origins of degradation mechanisms and a playbook of compositional, structural, and geometric design strategies to systematically alleviate degradation phenomena. In this perspective article, we discuss the chemical principles underpinning the coupling between electrochemical reactions, mechanics, and geometry across length scales with reference to positive electrode materials. The origins of the degradation of Li-ion battery performance are traceable to multi-field and multiphysics coupling initiated at atomistic scales manifested at mesoscale dimensions and compounded up to the level of electrode architectures. Such coupling mechanisms afford distinctive opportunities for design and impactful breakthroughs if they can be fundamentally understood and controlled. Data-driven materials holds promise for addressing this formidable challenge.

We discuss electrochemistry–mechanics coupling in positive electrode materials with regard to interfacial electrochemical reactions, bulk diffusion, and intercalation-induced phase transformations. Compositional heterogeneities during discharge/charge processes often give rise to dynamically evolving stress gradients, which can alter diffusion phenomena, even within limits of elastic deformation. Compounding stress gradients engender delamination, accumulation of misfit dislocations, granular fracture, and other modes of damage, which in turn further modify surface reactions, bulk diffusion, and phase separation. The interplay between mechanics and electrochemistry is governed both by intrinsic material properties as well as boundary conditions resulting from particle dimensions and mesoscale structure. We illustrate how tailoring of atomistic and electronic structure (materials design concepts), particle geometry, and particle mode of assembly can together or separately be used to alter electrochemistry–mechanics coupling across electrochemical reactions at the electrode surface, bulk diffusion, and phase transformations.

In this perspective, we've particularly emphasized generalizable design principles to unlock unexploited performance from new and existing battery chemistries, develop dynamic process controls, and design next-generation materials and architectures purpose-built to alleviate common modes of degradation. The design principles span the range from engineering crystallographic compatibility across phase transformations to the use of polymorphism to access precisely tunable diffusion paths and pre-intercalation to modify diffusion paths. Geometric design principles include control of particle dimensions and geometry, specific modes of assembly and clamping to enable coupling to external strain fields, and architected motifs. Many of the design principles discussed here overlap a few underlying mechanisms, suggesting that desired performance gains can be accessed from simultaneously implementing several strategies across multiple length scales. For example, phase transformations can be suppressed entirely by modifying the balance between the coherency strain penalty and the energetic preference for two-phase coexistence; this effect can either be achieved by tailoring the geometry of the active material or by raising the coherency strain penalty through atomistic modifications aimed at engineering lattice mismatch.

The deliberate design of electrode architectures to ensure long-term cycling requires a more accurate inventory of compositional inhomogeneities and stress gradients within active materials during battery operation. Advances in X-ray imaging and ultrafast X-ray methods are pushing the limits of spatial, temporal, and energy resolution, enabling an improved understanding of the spatiotemporal evolution of compositional heterogeneities and stress evolution. These measurements not only contribute to a fundamental understanding of degradation phenomena but further hold promise for the derivation of transfer functions from concurrently acquired electrochemical impedance spectroscopy data. Such functions will ideally manifest clearly distinguishable signatures of mechanical damage. The application of deep learning and dynamic meta-learning from different spaces can potentially allow for the prediction of damage thresholds being approached. Integration of anomaly detection, and even prediction, in battery management software holds promise for on-the-fly modification of operational conditions. A foundational understanding of electrochemistry–mechanics coupling, as emphasized in this work, can thus not only provide intrinsic materials design and assembly principles for alleviating degradation phenomena but also set the stage for dynamical process controls. An area that has attracted much attention is the design of dynamically transformable interfaces that can manifest morphogenesis and heal damage in ASSB architectures.

Altogether, the ability to unlock improved intercalation characteristics highlighted in this work emphasizes the importance of a paradigm shift from a purely electrochemical perspective on the design of electrodes to one that considers the rich and dynamic coupling between electrochemistry and mechanics. The close integration of chemical and physical processes is imperative to transcend rapidly emerging bottlenecks to TW h-scale energy storage applications.

## Author contributions

D. A. S. conceptualization, writing – original draft, writing – review & editing, visualization, supervision. S. R. conceptualization, writing – original draft, writing – review & editing. D. Z. writing – review & editing, visualization. Y. L. writing – original draft, investigation, formal analysis, visualization. B. L. writing – original draft. A. B. methodology, visualization, supervision. B-X. X. conceptualization, writing – review & editing, supervision and funding acquisition. S. B. conceptualization, writing – review & editing, supervision, funding acquisition.

## Conflicts of interest

There are no conflicts of interest to declare.

## Supplementary Material
